# Metabolic and enzymatic changes associated with carbon mobilization, utilization and replenishment triggered in grain amaranth (*Amaranthus cruentus*) in response to partial defoliation by mechanical injury or insect herbivory

**DOI:** 10.1186/1471-2229-12-163

**Published:** 2012-09-12

**Authors:** Paula Andrea Castrillón-Arbeláez, Norma Martínez-Gallardo, Hamlet Avilés Arnaut, Axel Tiessen, John Paul Délano-Frier

**Affiliations:** 1 Unidad de Biotecnología e Ingeniería Genética de Plantas (Cinvestav-Irapuato), Km 9.6 del Libramiento Norte Carretera Irapuato-León, Apartado Postal 629, C.P. 36821, Irapuato, Gto, México; 2 Present address: Instituto de Biotecnología, Facultad de Ciencias Biológicas, Universidad Autónoma de Nuevo León, Av. Pedro de Alba y Manuel L. Barragán s/n, Ciudad Universitaria, C.P. 66450, San Nicolás de los Garza, Nuevo León, México

**Keywords:** Carbohydrate metabolism, Carbohydrate mobilization, Carbon sequestration, Defoliation, Grain amaranth, Plasticity, Tolerance

## Abstract

**Background:**

*Amaranthus cruentus* and *A. hypochondriacus* are crop plants grown for grain production in subtropical countries. Recently, the generation of large-scale transcriptomic data opened the possibility to study representative genes of primary metabolism to gain a better understanding of the biochemical mechanisms underlying tolerance to defoliation in these species. A multi-level approach was followed involving gene expression analysis, enzyme activity and metabolite measurements.

**Results:**

Defoliation by insect herbivory (HD) or mechanical damage (MD) led to a rapid and transient reduction of non-structural carbohydrates (NSC) in all tissues examined. This correlated with a short-term induction of foliar sucrolytic activity, differential gene expression of a vacuolar invertase and its inhibitor, and induction of a sucrose transporter gene. Leaf starch in defoliated plants correlated negatively with amylolytic activity and expression of a β-amylase-1 gene and positively with a soluble starch synthase gene. Fatty-acid accumulation in roots coincided with a high expression of a phosphoenolpyruvate/phosphate transporter gene. In all tissues there was a long-term replenishment of most metabolite pools, which allowed damaged plants to maintain unaltered growth and grain yield. Promoter analysis of ADP-glucose pyrophosphorylase and vacuolar invertase genes indicated the presence of *cis*-regulatory elements that supported their responsiveness to defoliation. HD and MD had differential effects on transcripts, enzyme activities and metabolites. However, the correlation between transcript abundance and enzymatic activities was very limited. A better correlation was found between enzymes, metabolite levels and growth and reproductive parameters.

**Conclusions:**

It is concluded that a rapid reduction of NSC reserves in leaves, stems and roots followed by their long-term recovery underlies tolerance to defoliation in grain amaranth. This requires the coordinate action of genes/enzymes that are differentially affected by the way leaf damage is performed. Defoliation tolerance in grain is a complex process that can’t be fully explained at the transcriptomic level only.

## Background

The genus *Amaranthus* is comprised by more than 60 species. It belongs to the *Amaranthaceae* family that also includes sugar beet, spinach, *Chenopodium* spp. and several halophytes [1]. They are C_4_ dicotyledonous annual plants, many of which are ubiquitous weeds (e.g. *A. spinosus*, *A. tuberculatus* and *A. retroflexus*), whereas others (e.g. *A. tricolor* and *A. hybridus*), are used as foliar vegetables of high vitamin and mineral content, mainly in Asia and Africa [2,3]. They may be also a source of grain (mostly *A. caudatus*, *A. cruentus* and *A. hypochondriacus*). The latter are pseudo-cereals capable of producing seeds of high nutritive and nutraceutical value, granted by their optimal amino-acid balance, potential to release bioactive peptides upon digestion and relatively high squalene levels [3,4]. They are hardy plants with the ability to grow in poor soils or under unfavorable growing conditions, mostly involving low water availability, high salinity and/or high light intensity [3,5,6].

Vegetable and grain *Amaranthus* species have been reported to sustain high rates of infestation by herbivorous insects under field conditions, with differing effects on productivity [7,8]. Tolerance to defoliation in *A. hybridus* was associated with a greater investment in below-ground biomass relative to above-ground vegetative biomass, occurring mostly as the result of pre-flowering allocation of carbohydrates (CHOs) and nitrogen resources to the taproot [9,10]. Vegetable amaranths have been shown to recover exceptionally well from herbivore damage by grasshoppers and lepidopteran larvae [11]. However, certain insect pests can significantly reduce grain yield and increase the risk of lodging and infection by root and stem fungal pathogens [12-14]. Defoliation of grain amaranths by lepidopteran larvae at an early developmental stage has also been found to result in a long-term reduction in plant size and yield [2,15,16]. Insect infestation was more deleterious under drought-stress conditions [8]. Controlled experiments indicate that several *Amaranthus* species can fully recover from complete mechanical defoliation with small to negligible effects on fitness and yield (Vargas-Ortiz E, unpublished data). Moreover, mechanical removal of 10-to-40% of the primary shoot of grain amaranth plants is practiced in certain regions of Mexico to enhance secondary branching and biomass productivity [17].

Plants can respond to injury, including defoliation, by the deployment of a plethora of direct and/or indirect defenses [18,19]. However, when defenses are costly to produce or the resource demands for defense compete with those of growth and reproduction, damaged plants may undergo physiological changes such as the activation of dormant meristems, modified plant architecture, increased photosynthetic capacity, and/or the partitioning of resources among growth, storage, and reproduction, among others, in order to cope with the stress imposed by defoliation [20-22].

Source-sink relationship and carbon allocation in plants are regulated by complex metabolic and signaling networks [23]. Carbon levels in storage organs influence the net photosynthetic activity in source tissues, whereas the expression of photosynthesis-related enzymes in leaves is modified by sugar levels [24-26]. However, the mechanisms whereby sugars act to regulate source gene expression in C_4_ plants remain relatively unexamined [27].

Previous studies have focused on the defoliation responses of grain amaranth mostly in an ecological context. Here, we performed a more comprehensive study, involving a multifaceted approach, including genomic, promoter, gene expression and metabolite analyses in addition to enzyme activity assays. Two different defoliation treatments, insect herbivory (HD) and mechanical damage (MD), were tested considering that the responses to artificial defoliation can differ qualitatively and/or quantitatively from those produced by natural herbivory [see above; also [28,29]. The available genomic information of *Amaranthus hypochondriacus (Ah)*[30] was employed as a basis for gene selection and subsequent design of specific primers for real time PCR analysis and to isolate and characterize key genes involved in non-structural carbohydrate (NSC) metabolism. Gene expression at different times was studied in both sink and source tissues of damaged and control plants. The response depended strongly on the way defoliation was performed, as herbivory damage generally led to more intense changes in expression. The levels of many transcripts, enzymes and metabolites (hexoses, sucrose, starch and fatty acids) changed as a result of defoliation (e.g. short term decrease followed by a long-term recovery), thereby suggesting that tolerance to defoliation in amaranth might depend on the regulated use of NSC reserves controlled by key metabolic enzymes/genes. Thus, candidate grain amaranth genes known to influence C allocation and sink-source relationships in other plant models were analyzed in detail and some of them were cloned. However, the relationships between transcripts and enzymes were not always as straightforward as desired.

## Results and discussion

### Selection and cloning of Amaranth genes

Previous knowledge of primary metabolism and stress tolerance in other model species, and preliminary expression data in amaranth directed the selection a total of 25 genes. For a more detailed description see Additional file 1. In addition, seven cDNAs and two genomic sequences of key genes involved in carbon (C) partitioning were characterized (Tables 1 and 2). Two of the seven sucrose synthase (SuS) isoforms detected in the transcriptomic analysis [30] were chosen for further analysis. The phylogenetic analysis (Additional file 2) showed that AhSuS-1 and AhSuS-2 were highly homologous to the SBSS1-2 isoforms present in *Beta vulgaris*. These have been shown to be regulated by development and by several types of stress [31]. Plant invertases conform another multigenic family importantly involved in carbohydrate (CHO) mobilization. In grain amaranth, this family is represented by at least 19 isoforms. Two isoforms belonging to the alkaline neutral invertase subfamily, AhA/NI1-2, were selected for further study taking into account their similarity to invertases that are localized to the chloroplast; in wheat and Arabidopsis, their activity has been shown to be up-regulated by environmental stresses [32,33]. On the other hand, AhVI-1 was selected from a total of four vacuolar invertase isoforms on the basis of its high similarity to a vacuolar invertase in *B. vulgaris*, BvVI, which has been shown to play an important role in C partition [34]. The only putative cell wall invertase (CWI) isoform identified so far in graisfsffwr2n amaranth, AhCWI, is related to CWIs in *B. vulgaris* (BvExINV), *Vicia faba* (VfCWI2) and tomato (*Solanum lycopersicum*; SlWI1) which are known for their capacity to regulate sink strength in seeds and roots and participate in the wound response, respectively [34,35] (Additional file 3). The phylogenetic relationship between the ADP glucose pyrophosphorylases (AGP) found in grain amaranth suggests that the subunits that conforms the enzyme tetramer are being codified by at least four genes, two of which, AhAGP-L1 and −3 may be involved in stress responses. This is implied by their similarity to AGPL1-2 in tomato, which has been shown to be induced by salt stress in an ABA- and osmotic-stress independent way [36]. In contrast, only one small AGP subunit has been identified in grain amaranth, AhAGPS-1. This protein is related to homologs in other plants, such as tobacco, maize and pea, where they are known to participate in development programs and in stress-resistance responses [37-39] (Additional file 4). The partial sequence of six plant invertase inhibitors, which are small proteins (15–23 kD) targeted to the cell wall or vacuole in a variety of species [40], was also used to determine their phylogenetic relationships. The analysis showed that *AhInvI-4* had a close relationship to the Arabidopsis AtC/VIF-1, a confirmed vacuolar invertase inhibitor and that *AhInvI1-2* resembled apoplastic-localized inhibitors involved in both development (ZM-INVINH1) and stress response processes (AtC/VIF-1), respectively [41] (Additional file 5). The genomic sequences of a vacuolar invertase (*AhVI-1*) and of a plastidial ADP-glucose pyrophosphorylase small subunit gene (*AhAGPS-1*) included a sizeable section of their respective promoter regions. Promoter analysis of ADP-glucose pyrophosphorylase and vacuolar invertase genes indicated the presence of *cis*-regulatory elements that supported their responsiveness to defoliation (Additional files 6,7,8). Not surprisingly, the majority of the amaranth genes and proteins analyzed shared the highest level of identity with similar proteins previously reported in sugar beet, except for AhA/NI-1, which had a higher resemblance to an invertase isolated from carrot [42]. This coincided with the close phylogentic relationship shared by *Amaranthus* spp. and *B. vulgaris*[43,44]. An interesting aspect of the promoter regions of the *AhAGPS-1* gene was that it had a higher representation of regulatory elements involved in defense responses than that of an orthologous gene identified in *Solanum tuberosum*[45]*.* On the other hand, a striking difference found between the promoter regions of the *AhVI-I* and the *B. vulgaris* vacuolar invertase genes, respectively, was the lower abundance, in the former, of important cis-regulatory elements of genes involved in ABA and JA signaling pathways activated in response to (a)biotic stress and wounding (e.g. ABRE, G-box and W-box motifs) (Additional file 8). The expression of the selected genes was analyzed and correlated to the changes in carbohydrate (CHO) content and enzyme activities, as described in the following sections.

**Table 1:** cDNAs and predicted proteins of selected grain amaranth genes involved in sucrose and starch metabolism

**Gene**	**ORF**^**1**^**(bp)**^**2**^	**Size (bp)**^**3**^	**Size (KDa)**	**Identity (%)**	**Species**	**Accesion number**	**Salient characteristics**	**Ref**^**4**^
***AhAGPS-1***	1524	2019	55.7	91	*Beta vulgaris*	HM021763	The only small subunit AGPase detected in the *Ah*’s transcriptome. Highly similar to AGPase isoforms of sugar beet root and orange fruit (*Citrus sinensis*).	[46]
***AhSuS-1***	2412	2769	92.1	93	*Beta vulgaris*	JQ012918	Member of a small multi-gene family of at least seven different isoforms; similar to SBSS2 and CSS1proteins identified in roots of *B. vulgaris* and in *C. rubrum*, respectively. Classified within the *Dicot SUS-1*group.	[47-50]
***AhSuS-2***	2436	2832	93.3	78	*Beta vulgaris*	JQ012919	Similar to the SBSS1 protein induced by wounding, anoxia and cold-exposure in roots of *B*. *vulgaris*. Its putative mitochondrial localization implies novel roles apart from sucrose degradation. Classified within the *Dicot SUS-A* group.	[31,50-52]
***AhA/NI-1***	1671	1928	63.3	74	*Daucus carota*	JQ012920	An A/N invertase isoform predicted to be localized to the chloroplast (sub-clade β). Supports the general participation of A/NIs in the carbon flux between the cytosol and the plastids.	[53,54]
AhA/NI-2*	903	1171	———	77	*Vitis vinifera*	JQ012922	An A/N invertase isoform predicted to be localized to the chloroplast (sub-clade β).	[54,55]
***AhVI-1***	1977	2282	73.8	79	*Beta vulgaris*	JQ012921	Isoform grouped within the VI clade. Similar to a VI expressed in petioles of juvenile *B. vulgaris*, and to a soluble acid β-fructofuranosidase identified in *D. carota*. Its signal peptide predicts it to be a type II membrane protein that is anchored to the vacuolar membrane, similarly to Arabidopsis, rice, barley, and sugarcane. Membrane anchorage of AhVI-1 may permit a more precise control of its destination and activity.	[34,56,57]
AhCWI*	964	991	———	75	*Beta vulgaris*	JQ012923	Isoform grouped within the CWI clade. Has shared identity with CWIs from *B. vulgaris* and *C. rubrum.* Predicted to be a secretory protein having a hydrophobic 26 aa signal peptide that is required for co-translational insertion into the endoplasmic reticulum and secretion from the cell.	[34,48]

The percentage of identity with the closest annotated homolog is shown. cDNAs marked with an asterisk (*) are not full-length.

^1^ORF = Open Reading Frame.

^2^aa = amino-acids.

^3^ bp = base pairs.

^4^Ref = References.

**Table 2:** Genomic sequences of two grain amaranth genes involved in sucrose and starch metabolism

**Gene**	**Size (bp)**^**1**^	**No. Exon**	**No. Intron**	**Accesion number**	**Salient characteristics**^**2**^	**Ref**^**2**^
***AhAGPS-1***	5088	**9** (99–297)^3^	**8 (**84**–**1048)^3^	JQ034321	The gene is highly similar (94% identity) to the *B. vulgaris AGPB1* gene (GenBank X78899.1). The complexity of this gene is shared with other starch metabolism genes. The presence of a large first intron (1048 bp) suggests a possible role in regulating expression as observed for a sucrose synthase gene in Arabidopsis. The promoter region has MYCL and GCCF boxes which are needed in maize for the transcriptional regulation of the waxy gene coding for a GBSS.	[46,58-60]
***AhVI-1***	5376	**7** (9–857)^3^	**6** (85–1015)^3^	JQ012921	Contains the expected seven exons generally conserved in the majority of acid invertase genes isolated from plants^4^. The *AhVI-1* gene also contains a membrane spanning domain in exon 1 and the motifs NDPNG, partially encoded by mini-exon 2 encoding the tripeptide DPN, and WECVDF (exon 3), which are essential for catalytic activity and are conserved in this gene family^5^. A key feature identifying it as vacuolar invertase was that the X residue in the conserved WECXDF domain corresponded to a valine residue. This is characteristic of invertases targeted to the vacuole; in the CWIs, X is a proline.	[61-63]

The percentage of identity with the closest annotated homolog is shown.

^1^ bp = base pairs.

^2^Ref = Relevant references.

^3^Size range (bp).

^4^Refer to Additional file 6A.

^5^Refer to Additional files 6B and 6C.

### Changes in CHO levels produced in response to partial defoliation in *A. cruentus*

Partial defoliation (≈ 30% loss of leaf tissue) was produced in 30-day-old plants either by mechanical damage (mechanical defoliation, MD) or insect feeding (herbivory defoliation, HD). Measurements were made at different days *post* partial defoliation (dppd): 1, 5, 30 and 110 dppd in three independent experiments. The choice of these time points was based on preliminary experiments [15,16].

**Figure 1:**
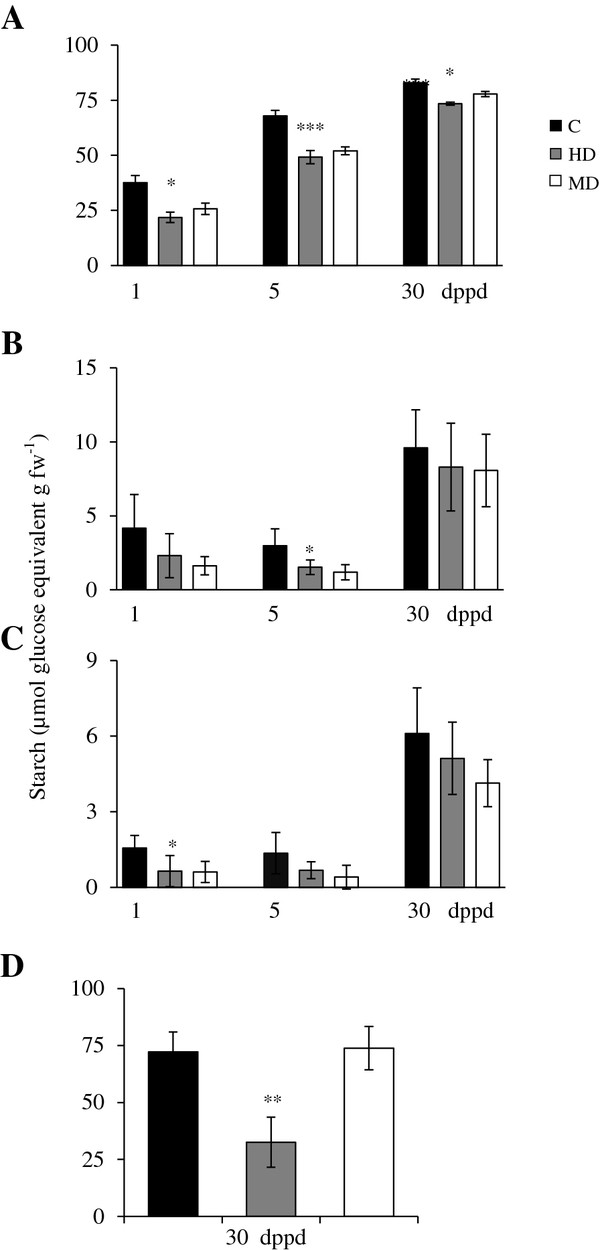
**Starch levels measured as glucose equivalents per g of fresh weight at different days post partial defoliation (dppd) in (A) source leaves**^**1**^**, (B) stems, (C) roots and (D) panicles of intact control and defoliated*Amaranthus cruentus*plants.** Defoliation was produced either by insect herbivory (HD) or mechanical damage (MD). Data represent means ± standard error of three technical replicates of pooled samples taken from a representative experiment that was replicated twice. Asterisks indicate significant difference from controls at *P < 0.05; **P < 0.01; ***P < 0.001. ^1^In defoliated plants, all three source leaves sampled were damaged.

**Figure 2:**
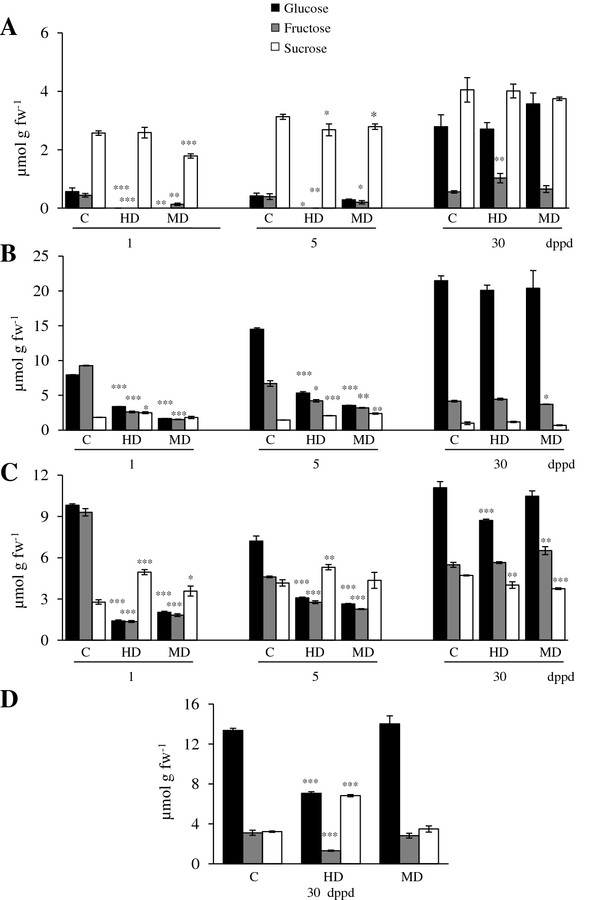
**Sucrose, glucose and fructose levels measured at different days post partial defoliation (dppd) in (A) source leaves, (B) stems, (C) roots and (D) panicles of intact control and defoliated *Amaranthus cruentus *plants. **Defoliation was produced either by insect herbivory (HD) or mechanical damage (MD). For further details refer to Figure 1.

Starch, SUC, GLC and FRC levels were determined in source leaves, stems, roots and panicles of *A. cruentus* (*Ac*) plants (Figures 1 and 2). The general trend was that all non-structural carbohydrate (NSC) levels were reduced in most tissues as a result of defoliation (Figures 1 and 2). Starch and hexoses were the NSCs more profoundly affected by defoliation. Starch was predominantly reduced in leaves of MD and HD plants compared to undamaged controls (Figure 1A), whereas hexoses were depleted in leaves, stems and roots of MD and HD plants compared to undamaged controls (Figure 2). The effect was rapid, since it occurred most dramatically at 1 dppd and was still evident at 5 dppd (Figures 1 and 2). Hexoses depletion at times when sucrolytic activities were high (e.g. 5 dppd; see below) suggested that their utilization rate in the defoliated plants surpassed their enzymatic release rate from sucrose. The rapid and general reduction in NSC reserves occurring in response to defoliation was very similar to the one observed when plants were C-starved by shading for three consecutive days (Vargas-Ortiz E, unpublished data). This similarity supports our proposal that foliar starch and other C reserves are rapidly metabolized to sustain growth when they are depleted by factors that affect C acquisition in leaves, such as reduced leaf area or light limitation.

On the long term most NSC levels recovered or maintained control levels at 30 dppd, and in some isolated cases surpassed them (Figure 2). It was also evident that MD and HD led to similar changes in NSC levels, except in panicles, where HD had a strong negative effect in all NSCs except SUC (Figures 1 and 2).

Starch was the most abundant NSC reserve in the leaves of *Ac* plants (Figure 1). This agreed with the high starch accumulation in leaves of sugar beet, a close relative of amaranth [64]. Starch levels in leaves and panicles were ~10 times higher than roots and stems (Figure 1). Starch increased approximately 2-fold during development, but the HD and MD treatments consistently decreased starch levels compared to the control plants (Figure 1). This pattern suggested that the mobilization of the ample starch reserves in leaves of *Ac* could be a key factor for recovery after defoliation. It was in agreement with the frequently observed mobilization of starch reserves to sustain new plant growth after defoliation and with the proposed role of leaf and stem starch as a buffer against abiotic and biotic stresses [65]. Curiously, these results had a closer resemblance to the long-term responses to defoliation reported in woody plants than to those registered in annual grasses and shrubs. In the former species, defoliation has been frequently found to cause short-term reductions in C stores, present primarily in leaves, but no long term depletion after repeated defoliation [66-70]. On the other hand, seasonal defoliation studies performed with C_3_ grasses (e.g. *Agropyron spp.* and *Lolium perenne*) and shrubs (e.g. *Caragana korshinskii* and *Ruellia nudiflora*), have reported more diverse outcomes. These include, in *L. perenne*, an accumulation of C reserves that was subsequently used to secure survival after repeated grazing [71]. In contrast, a drastic reduction in soluble carbon pools was detected in *Agropyron* bunchgrasses after severe defoliation treatments that impeded full recovery due to a limited replenishment capacity [72]. In shrubs, reduced leaf longevity and accumulation of below-ground carbon reserves allowed full compensation in terms of fruit output in defoliated *R. nudiflora*[73], whereas *C. korshinskii* relied on the preferential resource allocation to vegetative tissues for regrowth, at the cost of fruit production [74].

It must be noted that in one experiment, in which starch levels in leaves were 2-fold higher than usual, MD led to a 2-to-3 fold increase of starch in stems and roots at 30 dppd (Additional file 9B and C). This suggests a plastic response to defoliation in *Ac* plants, which can sequester C in less vulnerable storage tissues when favorable growth conditions permit the accumulation of high foliar starch. Also, the tissue- and ontogenetic dependent changes in NSC levels observed (Figures 1, 2), implied that C resources can be preferentially allocated to certain tissues at certain developmental stages to be used for growth, reproduction or, perhaps, defense. This raised the question of which enzymes could be regulating C allocation in grain amaranth.

### Changes in SuS activities and expression in response to partial defoliation in *A. cruentus*

The observed fluctuations in NSC levels in sink and source tissues in response to partial defoliation (Figure 2) could have been caused by changes in different sucrolytic activities present in different subcellular compartments and tissues. Therefore, the activities of SuS, neutral invertases, soluble acid and cell-wall-bound invertases were determined at different times after HD or MD. The enzymatic assays were complemented with a qPCR expression analysis of selected isoforms and genes associated with C allocation and transport (Tables 3,4,5,6).

**Table 3:** Relative transcript abundance of sucrolytic and related regulatory genes

***Gene***^***1***^	***Treatment***	***dppd***	***Leaf***	***Stem***	***Root***	***Panicle***
***AhSuS-1***	HD	1	***0.18 ± 0.00***	1.50 ± 0.20	**2.09 ± 0.10**	—
		5	1.38 ± 0.05	0.87 ± 0.21	0.83 ± 0.08	—
		30	**3.01 ± 0.24**	**2.15 ± 0.40**	1.03 ± 0.19	**2.15 ± 0.08**
	MD	1	0.63 ± 0.06	NE	0.95 ± 0.16	—
		5	0.87 ± 0.07	0.84 ± 0.07	0.53 ± 0.08	—
		30	1.23 ± 0.27	1.06 ± 0.30	0.55 ± 0.01	1.47 ± 0.35
***AhSuS-2***	HD	1	***0.36 ± 0.03***	**2.10 ± 0.10**	**1.64 ± 0.21**	—
		5	1.21 ± 0.02	0.77 ± 0.09	**1.52 ± 0.03**	—
		30	**2.62 ± 0.07**	**1.58 ± 0.14**	**1.77 ± 0.25**	**1.91 ± 0.05**
	MD	1	0.76 ± 0.04	**2.36 ± 0.41**	1.19 ± 0.10	—
		5	0.86 ± 0.08	0.66 ± 0.01	0.86 ± 0.23	—
		30	1.13 ± 0.08	**2.00 ± 0.21**	1.10 ± 0.06	1.07 ± 0.04
***AhVI-1***	HD	1	**2.19 ± 0.18**	**3.00 ± 0.90**	1.37 ± 0.13	—
		5	**3.74 ± 0.00**	**6.58 ± 0.26**	**2.49 ± 0.05**	—
		30	0.91 ± 0.25	1.12 ± 0.15	1.20 ± 0.18	1.38 ±0.02
	MD	1	0.82 ± 0.01	1.25 ± 0.06	**1.96 ± 0.14**	—
		5	**2.57 ± 0.06**	**2.71 ± 0.76**	1.30 ± 0.43	—
		30	1.24 ± 0.04	1.11 ± 0.13	0.77 ± 0.06	**1.65 ± 0.04**
***AhInvI-4*(VI inhibitor)**	HD	1	***0.40 ± 0.17***	1.00 ± 0.30	**1.54 ± 0.11**	—
		5	1.00 ± 0.01	0.89 ± 0.34	0.78 ± 0.22	—
		30	**1.77 ± 0.29**	0.60 ± 0.06	0.72 ± 0.18	1.30 ± 0.22
	MD	1	0.68 ± 0.40	1.12 ± 0.39	0.86 ± 0.09	—
		5	0.96 ± 0.06	0.61 ± 0.34	0.61 ± 0.16	—
		30	**2.89 ± 0.53**	**1.86 ± 0.04**	0.98 ± 0.10	0.76 ± 0.09
***Ah-γVPE***	HD	1	**4.40 ± 0.54**	0.70 ± 0.10	1.41 ± 0.19	—
		5	***0.10 ± 0.01***	**1.91 ± 0.25**	**2.74 ± 0.52**	—
		30	**5.83 ± 0.92**	0.98 ± 0.30	**1.61 ± 0.13**	**2.09 ± 0.11**
	MD	1	***0.49 ± 0.20***	0.59 ± 0.27	1.15 ± 0.11	—
		5	***0.35 ± 0.04***	***0.39 ± 0.12***	***0.13 ± 0.01***	—
		30	**3.59 ± 0.01**	1.10 ± 0.55	1.39 ± 0.14	**1.76 ± 0.09**
***AhInvI-2*(CWI inhibitor)**	HD	1	***0.23 ± 0.02***	1.40 ± 0.20	**1.51 ± 0.23**	—
		5	**1.59 ± 0.14**	0.85 ± 0.06	0.75 ± 0.10	—
		30	**1.94 ± 0.28**	1.12 ± 0.02	0.97 ± 0.03	1.39 ± 0.16
	MD	1	***0.35 ± 0.19***	**2.45 ± 1.69**	1.29 ± 0.16	—
		5	1.30 ± 0.00	0.56 ± 0.04	1.00 ± 0.06	—
		30	0.88 ± 0.01	**2.56 ± 0.15**	0.94 ± 0.16	1.26 ± 0.10
***AhInvI-1*(CWI inhibitor)**	HD	1	***0.20 ± 0.03***	^2^NE	***0.29 ± 0.06***	—
		5	0.53 ± 0.09	0.96 ± 0.01	0.98 ± 0.00	
		30	1.03 ± 0.13	0.91 ± 0.16	0.70 ± 0.03	1.05 ± 0.13
	MD	1	0.70 ± 0.05	NE	1.22 ± 0.16	—
		5	0.63 ± 0.04	1.19 ± 0.51	**1.86 ± 0.54**	—
		30	0.94 ± 0.22	**2.48 ± 0.23**	**2.08 ± 0.19**	0.93 ± 0.15
***AhA/NI-1***	HD	1	***0.09 ± 0.20***	0.79 ± 0.26	NE	—
		5	0.94 ± 0.37	**3.59 ± 0.31**	NE	—
		30	NE	1.38 ± 0.16	NE	NE
	MD	1	***0.11 ± 0.20***	**1.64 ± 0.06**	NE	—
		5	***0.22 ± 0.47***	**6.10 ± 0.22**	NE	—
		30	NE	0.80 ± 0.17	NE	NE
***AhSNRK-1***	HD	1	**1.87 ± 0.05**	—	—	—
		5	1.29 ± 0.38	—	—	—
		30	***0.40 ± 0.27***	—	—	—
	MD	1	***0.18 ± 0.16***	—	—	—
		5	**4.70 ± 0.50**	—	—	—
		30	**3.87 ± 0.27**	—	—	—

Expression was measured at different days *post* partial defoliation (dppd) by insect (HD) or mechanical (MD) damage in different plant tissues of *Amaranthus cruentus*. Transcript levels were measured by qRT-PCR. Values represent means ± SE of a representative experiment with three replicates. They indicate the fold-change in gene expression levels present in tissues of defoliated plants relative to those in tissues of intact control plants. A given gene was considered induced when its relative expression values was ≥ 1.5, whereas it was considered repressed when its relative expression values was ≤ 0.5. These are shown in bold text and bold italics, respectively.

^1^The expression of the neutral invertase *AhA/NI-2* and sucrose phosphate synthase (*AhSPS*) genes was not detected in any of the tissues examined.

^2^NE = Not Expressed.

**Table 4:** Relative transcript abundance of genes involved in starch biosynthesis or breakdown

***Gene***^***1***^	***Treatment***	***dppd***	***Leaf***	***Stem***	***Root***	***Panicle***
***AhAGPS-1***	HD	1	0.99 ± 0.38	0.80 ± 0.11	1.24 ± 0.13	—
		5	1.17 ± 0.11	***0.49 ± 0.09***	0.59 ± 0.07	—
		30	0.65 ± 0.25	***0.31 ± 0.13***	0.74 ± 0.04	1.30 ± 0.14
	MD	1	1.06 ± 0.47	**1.88 ± 0.57**	1.25 ± 0.14	—
		5	**1.68 ± 0.23**	1.04 ± 0.24	1.08 ± 0.29	—
		30	0.69 ± 0.04	1.12 ± 0.25	1.36 ± 0.21	1.13 ± 0.18
***AhAGPL-1***	HD	1	***0.17 ± 0.02***	^2^NE	1.14 ± 0.16	—
		5	0.87 ± 0.15	0.85 ± 0.25	0.86 ± 0.22	—
		30	1.10 ± 0.12	0.74 ± 0.00	1.02 ± 0.09	0.87 ± 0.33
	MD	1	***0.40 ± 0.01***	1.19 ± 0.02	0.80 ± 0.11	—
		5	0.77 ± 0.17	***0.49 ± 0.11***	0.55 ± 0.13	—
		30	0.53 ± 0.01	0.97 ± 0.18	0.55 ± 0.08	0.74 ± 0.16
***AhAGPL-2***	HD	1	1.31 ± 0.13	0.90 ± 0.00	**2.08 ± 1.02**	—
		5	**1.98 ± 0.10**	**1.87 ± 0.37**	**3.12 ± 0.27**	—
		30	0.68 ± 0.22	1.36 ± 0.16	**2.11 ± 0.51**	**2.19 ± 0.11**
	MD	1	0.92 ± 0.15	1.29 ± 0.35	1.12 ± 1.06	—
		5	1.46 ± 0.18	1.16 ± 0.25	0.88 ± 0.21	—
		30	0.88 ± 0.01	**2.56 ± 0.15**	0.94 ± 0.16	1.26 ± 0.10
***AhAGBSS***	HD	1	1.10 ± 0.18	0.64 ± 0.10	NE	—
		5	0.92 ± 0.05	0.55 ± 0.33	NE	—
		30	***0.35 ± 0.00***	0.95 ± 0.05	NE	1.00 ± 0.31
	MD	1	1.22 ± 0.17	0.61 ± 0.19	NE	—
		5	***0.47 ± 0.28***	0.54 ± 0.13	NE	—
		30	0.55 ± 0.99	1.27 ± 0.12	NE	0.56 ± 0.67
***AhSS-IV***	HD	1	***0.17 ± 0.38***	—	—	—
		5	0.92 ± 0.54	—	—	—
		30	**39.64 ± 0.60**	—	—	—
	MD	1	1.38 ± 0.08	—	—	—
		5	0.68 ± 0.06	—	—	—
		30	**9.59 ± 0.18**	—	—	—
***AhBMY-1***	HD	1	**2.10 ± 0.45**	—	—	—
		5	***0.44 ± 0.37***	—	—	—
		30	NE	—	—	—
	MD	1	0.78 ± 0.64	—	—	—
		5	**12.12 ± 0.31**	—	—	—
		30	NE	—	—	—

Expression was measured at different days *post* partial defoliation (dppd) by insect (HD) or mechanical (MD) damage in different plant tissues of *Amaranthus cruentus*. Refer to Table 3 for further details.

^1^The expression of the soluble starch synthase *AhSS-III* genes was not detected in any of the tissues examined.

^2^NE = Not Expressed.

**Table 5:** Relative transcript abundance of transport genes associated with carbohydrate metabolism and/or sink-source relationships

***Gene***^***1***^	***Treatment***	***dppd***	***Leaf***	***Stem***	***Root***	***Panicle***
***AhSUT-1***	HD	1	**3.04 ± 0.03**	^2^NE	1.18 ± 0.14	—
		5	1.11 ± 0.01	0.63 ± 0.10	***0.32 ± 0.04***	—
		30	0.90 ± 0.12	**1.59 ± 0.10**	1.16 ± 0.05	**1.58 ± 0.08**
	MD	1	1.23 ± 0.10	NE	0.68 ± 0.00	—
		5	0.69 ± 0.01	0.69 ± 0.05	0.63 ± 0.03	—
		30	0.70 ± 0.00	1.05 ± 0.00	0.57 ± 0.01	1.11 ± 0.03
***AhPPT***	HD	1	***0.15 ± 0.06***	0.79 ± 0.25	1.15 ± 0.13	—
		5	**2.03 ± 0.05**	***0.23 ± 0.21***	**22.09 ± 0.64**	—
		30	1.28 ± 0.61	1.09 ± 0.04	1.43 ± 0.64	0.88 ± 0.03
	MD	1	1.44 ± 0.27	1.28 ± 0.12	**2.32 ± 0.08**	—
		5	**1.91 ± 0.05**	**2.54 ± 0.31**	**25.45 ± 1.29**	—
		30	***0.35 ± 0.03***	0.94 ± 0.11	**6.86 ± 0.51**	0.73 ± 0.29

Expression was measured at different days *post* partial defoliation (dppd) by insect (HD) or mechanical (MD) damage in different plant tissues of *Amaranthus cruentus*. Refer to Table 3 for further details.

^1^The expression of the Glucose-6-phosphate transporter *AhG6PT* gene was not detected in any of the tissues examined.

^2^NE = Not Expressed.

**Table 6:** **Relative transcript abundance of wound-response- (*AhKTI*), jasmonic acid- (*AhLOX2*) and senescence/ development- (*AhSAG*) marker genes**

***Gene***	***Treatment***	***dppd***	***Leaf***	***Stem***	***Root***	***Panicle***
***AhKTI***	HD	1	**5.18 ± 0.70**	0.90 ± 0.20	**2.04 ± 0.51**	—
		5	**6.40 ± 0.69**	0.87 ± 0.02	1.06 ± 0.06	—
		30	0.77 ± 0.19	1.44 ± 0.03	1.08 ± 0.12	**1.54 ± 0.30**
	MD	1	**1.66 ± 0.85**	**6.40 ± 0.10**	0.94 ± 0.23	—
		5	**3.22 ± 0.10**	1.19 ± 0.36	**1.92 ± 0.36**	—
		30	***0.20 ± 0.03***	**2.49 ± 0.17**	0.74 ± 0.25	1.46 ± 0.24
***AhLOX2***	HD	1	**5.39 ± 0.53**	0.89 ± 0.10	0.59 ± 0.29	—
		5	**2.09 ± 0.00**	***0.14 ± 0.10***	^1^NE	—
		30	**1.59 ± 0.03**	1.22 ± 0.15	NE	1.36 ± 0.04
	MD	1	0.71 ± 0.83	1.18 ± 0.16	**1.51 ± 0.46**	—
		5	0.97 ± 0.03	***0.24 ± 0.03***	NE	—
		30	1.29 ± 0.18	1.46 ± 0.03	NE	0.75 ± 0.08
***AhSAG***	HD	1	***0.10 ± 0.04***	NE	**1.58 ± 0.48**	—
		5	**1.80 ± 0.41**	0.56 ± 0.18	0.84 ± 0.01	—
		30	**4.38 ± 0.13**	NE	1.07 ± 0.11	**26.0 ± 0.00**
	MD	1	0.70 ± 0.05	***0.12 ± 0.00***	**2.02 ± 0.32**	—
		5	1.27 ± 0.01	0.73 ± 0.25	1.14 ± 0.07	—
		30	1.41 ± 0.02	**1.55 ± 0.05**	1.03 ± 0.10	1.28 ± 0.16

Expression was measured at different days *post* partial defoliation (dppd) by insect (HD) or mechanical (MD) damage in different plant tissues of *Amaranthus cruentus*. Refer to Table 3 for further details.

^1^NE = Not Expressed.

**Figure 3:**
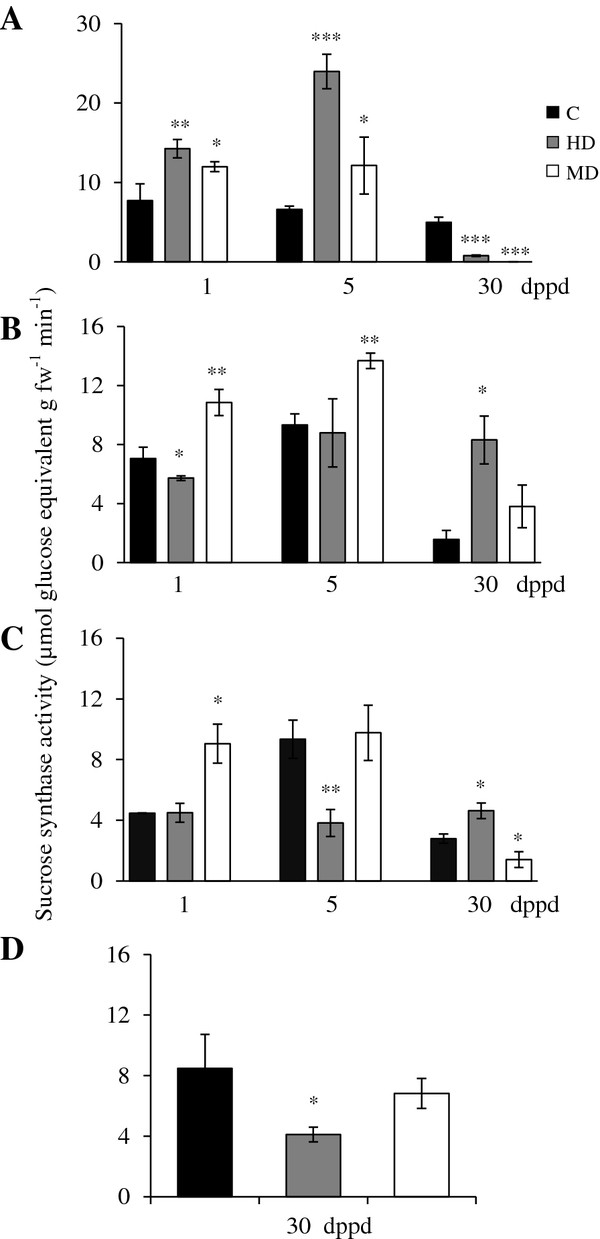
**Sucrolytic sucrose synthase activity levels measured as μmoles of glucose equivalents per g of fresh weight released per minute at different days post partial defoliation (dppd) in (A) source leaves, (B) stems, (C) roots and (D) panicles of intact control and defoliated *Amaranthus cruentus *plants. ** Defoliation was produced either by insect herbivory (HD) or mechanical damage (MD). For further details refer to Figure 1.

SuS activity in undamaged plants remained relatively stable for the duration of the defoliation experiment, except for transient peaks in activity in stems and roots of 35 day-old plants (equivalent to the 5 dppd in defoliated plants) (Figure 3). Defoliation had an evident effect on SuS activity (Figure 3). For example, in leaves, both MD and HD induced a 2- to 4-fold increase in SuS activity at 1 and 5 dppd (Figure 3A). The effect was reversed at 30 dppd, where both treatments led to a drastic reduction of SuS activity (Figure 3A). The changes of SuS activity might have reflected a shift in the metabolic status of leaves, from sink to source tissues, as they gradually recovered their NSC reserves, as observed in Figures 1 and 2.

**Figure 4:**
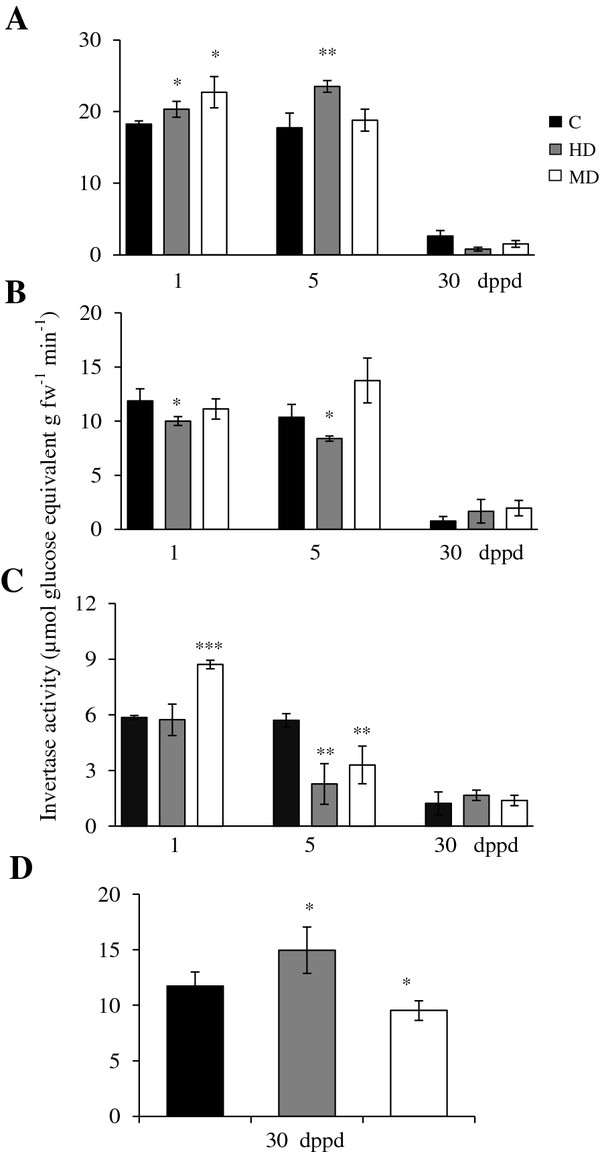
**Soluble acid (*vacuolar***) **invertase (SAI) activity levels measured as μmoles of glucose equivalents per g of fresh weight released per minute at different days post partial defoliation (dppd) in (A) source leaves, (B) stems, (C) roots and (D) panicles of intact control and defoliated *Amaranthus cruentus *plants. ** Defoliation was produced either by insect herbivory (HD) or mechanical damage (MD). For further details refer to Figure 1.

The *AhSuS-1* and *AhSuS-2* genes were more sensitive to HD than to MD (Table 3). The latter treatment only affected the expression of these genes in stems, where they showed a contrasting behavior (Table 3; see below). Moreover, no concordance was found between the biphasic pattern of *AhSuS* gene expression in leaves of HD plants (low at 1dppd and high at 30 dppd) and enzyme activity levels, which were higher than controls shortly after defoliation and then dropped to almost undetectable levels at 30 dppd.

In stems of MD plants, SuS activity was temporarily induced above those in undamaged controls at 1 and 5 dppd. HD, in contrast, led to a rapid decline at 1 dppd, but maintained a long-term stability that led to higher activity levels than controls at 30 dppd (Figure 3B). The significantly higher SuS activity that persisted in stems of HD plants at 30 dppd coincided with the late induction of both *AhSuS-1* and *AhSuS-2* (Table 3). In roots, MD had a fast inductive effect on SuS activity that was not accompanied by the induction of either of the *AhSuS-1* and *AhSuS-2* genes. Similarly, the ~2-fold reduction in SuS activity in panicles of HD plants was contrary to the induced expression of both *AhSuS* genes in these tissues in HD plants (Figure 3D and Table 3). The lack of direct correlation observed between *AhSuS-1* and *AhSuS-2* expression levels and SuS activity resembled the disparity observed between SuS gene expression and enzyme activity in sugar beet roots subjected to wounding or anoxia, which was attributed to post-transcriptional mechanisms [31,75]. The discrepancy between SuS activity and expression could also be explained by the participation of other putative *SuS* isoforms. Similarly to various other plant species [76], *SuS* genes in grain amaranth conform a multi-gene family consisting at least seven different members [30].

The systemic induction of both *AhSuS* genes and activity in stems, roots and/ or panicles of defoliated plants was consistent with the frequent induction of *SuS* genes in roots and/or shoots of plants under stress conditions [77]. This argues in favor of the generation of a wound-derived systemic signal in defoliated amaranth plants, which could have been JA, a related oxylipin or even H_2_O_2_ (see below).

### Changes in invertase activities and expression in response to partial defoliation in *A. cruentus*

Soluble acid invertase (SAI) activity (e.g. “vacuolar invertase”) was comparatively high in leaves of damaged young *Ac* plants (31 and 35 days-old-plants), intermediate in stems and low in roots (Figure 4A-C). SAI activity declined sharply in all tissues of 60 days-old-plants (30 dppd), except in panicles where SAI activity levels were similar to those detected in stems of young plants (Figure 4D). In leaves, the defoliation effect on SAI was very similar to the one observed on SuS activity (except that no induction was observed at 5 dppd in MD plants) (Figure 4A). In stems, the short term effect of HD, at 1 and 5 dppd, was negative, whereas MD was neutral (Figure 4B). In roots, the effect of defoliation was negative at 5 dppd, although a transient induction was observed in MD plants at 1 dppd, whereas increased SAI activity was detected in panicles of HD plants (Figure 4C and D).

**Figure 5:**
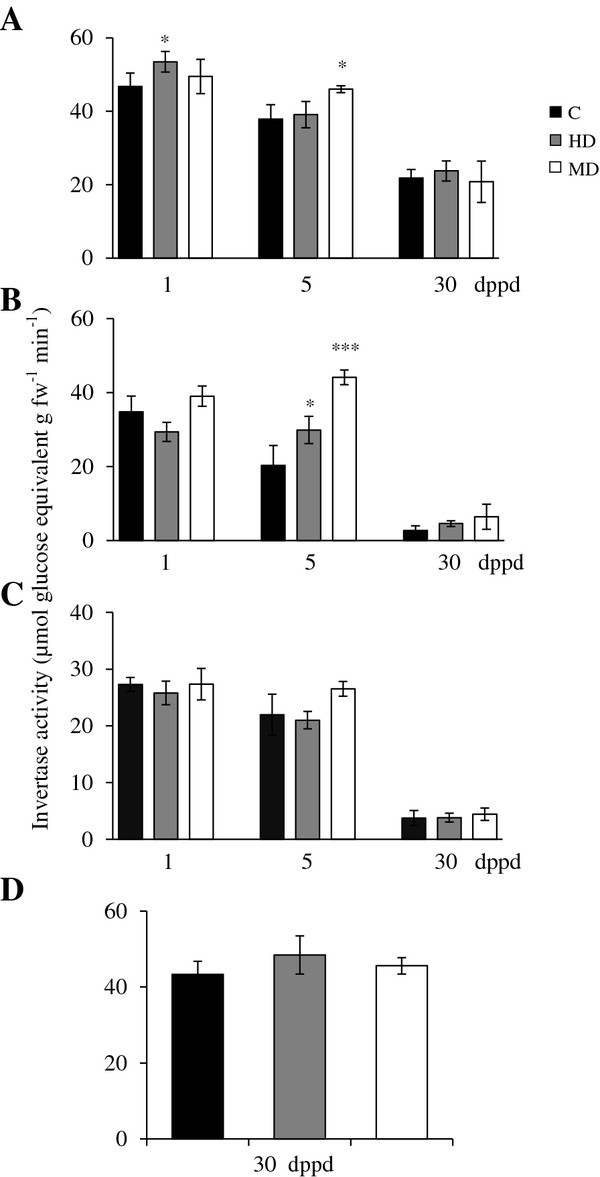
**Insoluble acid (*cell wall*) invertase (IAI) activity levels measured as μmoles of glucose equivalents per g of fresh weight released per minute at different days post partial defoliation (dppd) in (A) source leaves, (B) stems, (C) roots and (D) panicles of intact control and defoliated *Amaranthus cruentus *plants.** Defoliation was produced either by insect herbivory (HD) or mechanical damage (MD). For further details refer to Figure 1.

*AhVI-1* expression could explain the increase of SAI activity in leaves of HD, at 1 and 5 dppd, and MD plants, at 5 dppd, and in stems of MD plants, at 5 dppd (Table 3). In addition, the late induction of the putative *AhInvI-4* inhibitor gene predicted to target AhVI-1 [41] (Additional file 5) correlated with the abated SAI activity levels observed in leaves of HD and MD plants at 30 dppd (Table 3 and Figure 4A). The repressed expression of this inhibitor gene observed in leaves at 1 dppd could have also contributed to the increased SAI activity detected.

The expression of a vacuolar processing enzyme gamma gene (*Ah-γVPE*) in response to defoliation was also monitored in this study because of its known participation in the regulation of vacuolar invertase activity *in planta*[56,78,79]. However, only its late induction in leaves in response to both HD and MD, at 30 dppd, and its early and strong repression in MD plants, at 1 dppd, coincided with modified SAI activity levels (Table 3 and Figure 4). Curiously, a strong repression of this gene was observed in leaves of defoliated plants at 5 dppd. Apart from leaves, an evident damage-dependent effect on the expression of this gene was observed at this time point in stems and roots, being positive in HD plants and negative in MD plants. The general lack of correlation between *Ah-γVPE* expression and SAI levels in *Ac* indicated other possible functions of this gene in stressed plants, similar to what was recently reported in *Nicotiana benthamiana*[80].

Increased *AhVI-1* transcript abundance and acid invertase activity in damaged leaves and sink tissues of defoliated *Ac* plants was in accordance with the known up-regulation of invertases often observed after leaf damage by wounding or insect herbivory in several plant species, where they are believed to contribute to the altered source-sink relationships occurring in damaged leaves [21,35,81,82]. Moreover, the pattern of *AhVI-1* expression and SAI activity suggest that this particular sucrolytic enzyme, perhaps in combination with SuS activity (see above), may play an important role in the early utilization of C reserves, predominantly in leaves, to support growth under defoliation stress in *Ac* plants.

Insoluble (e.g. “cell wall-bound”) acid invertase (IAI) activity was, in general, the most active type of invertase detected in *Ac* plants. IAI levels were similar in leaves and stems and lower in roots, of undamaged young *Ac* plants and diminished in these tissues as plants aged. IAI activity in young panicles was relatively high (Figure 5). The defoliation effect on IAI activity in leaves was sporadic, only induced in leaves by HD and MD at 1 and 5 dppd, respectively, and in stems, at 5 dppd (Figure 5A and B). These minor changes in CWI activity were mirrored by no apparent changes in *AhCWI* gene expression levels (results not shown).

*AhInvI-2* (coding for a putative inhibitor of CWIs) showed widely different patterns of expression (Table 3 and Figure 5). The expression of this inhibitor gene in leaves of HD plants also showed an inverse correlation with the pattern of CWI activity (Figure 5A and Table 3), since its down-regulation, at 1 dppd, correlated with augmented levels of CWI activity. The *AhInvI-1* gene, coding for another putative CWI inhibitor, also showed tissue- and damage-dependent expression, since HD led to strong and transient repression at 1 dppd in all tissues examined, whereas MD mostly led to a retarded to late induction, at 5 and 30 dppd, in stem and leaves. However, the target enzymes and physiological roles of these particular inhibitors in SUC metabolism in *Ac* plants remain to be determined.

**Figure 6:**
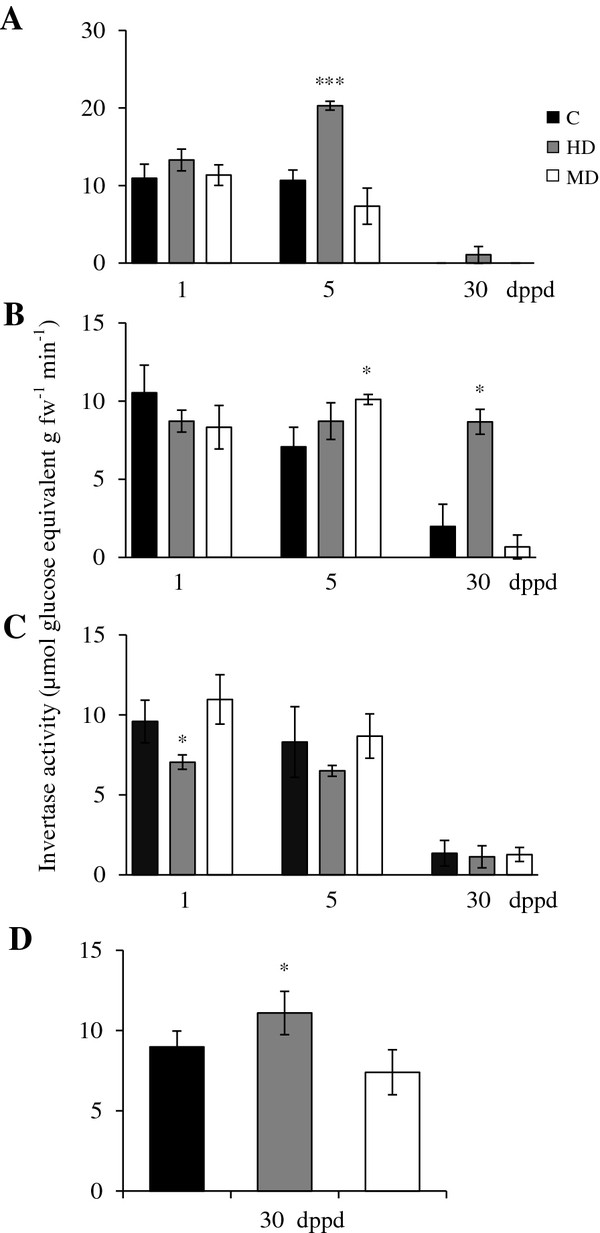
**Soluble neutral (*cytoplasmic*) invertase (NI) activity levels measured as μmoles of glucose equivalents per g of fresh weight released per minute at different days post partial defoliation (dppd) in (A) source leaves, (B) stems, (C) roots and (D) panicles of intact control and defoliated *Amaranthus cruentus *plants. ** Defoliation was produced either by insect herbivory (HD) or mechanical damage (MD). For further details refer to Figure 1.

Neutral (e.g. “cytoplasmic”) invertase (NI) was, in general, the least active type of invertase detected in *Ac* plants. NI activity levels did not vary much at first in all tissues of undamaged young *Ac* plants examined, but similarly to SAI and IAI, NI activity declined sharply in 60 days-old-plants, reaching undetectable levels in leaves (Figure 6). This coincided with the low to undetectable levels of expression of the neutral invertase *AhA/NI-1* gene in leaves and roots of control and defoliated plants at 30 dppd (Table 3). Similar to other sucrolytic enzymes, the development-related decrease in NI activity was not observed in panicles, where levels remained similar to those detected in younger vegetative tissues (Figure 6D). However, the gene expression assays indicated that the persistent NI activity observed in panicles was not due to the *AhA/NI-1* gene, where the expression of this gene was not detected. On the other hand, the strong up-regulation of this gene at 5 dppd in stems of HD and MD plants coincided only with the significantly higher NI activity levels in MD stems. No such correlation was observed in older stems, where the unexpectedly high activity detected at 30 dppd in HD plants, was not accompanied by a concomitant increase in *AhA/NI-1* expression (Figure 6B and Table 3). The *AhA/NI-1* expression pattern observed suggested a rigid tissue-specificity, since it was not detected in roots and panicles, was mostly down-regulated in leaves, and was strongly induced in stems at 5 dppd. This likewise suggests the participation of other *AhA/NI* genes in determining defoliation-induced changes in alkaline/ neutral invertase activity in the cytoplasm, and possibly in plastids and/ or mitochondria [83]. This is in agreement with the detection of at least 14 other putative NI isoforms in grain amaranth [30].

### Genes of starch metabolism in response to partial defoliation in *A. cruentus*

The expression of *AhAGPS-1* and *AhGBSS* was mostly unaffected or down-regulated by defoliation (Table 4). Moreover, no expression of the soluble starch synthases class III gene (*AhSS-III*) was detected. In contrast, the two *AhAGPL* genes examined in this study, showed completely different patterns of expression in response to defoliation, both with respect to *AhAGPS-1* and to each other (Table 4). Defoliation had a neutral to negative effect on the expression of *AhAGPL-1*, and was most strongly manifested at the early stages after defoliation (1 and 5 dppd) in leaves and stems of damaged plants. In contrast, *AhAGPL-2* was shown to be highly sensitive to HD plants in a time- and tissue dependent manner, as manifested by its induced expression in all tissues examined, particularly at 5 dppd, and its consistent up-regulation in roots at all times after HD. MD was a weaker stimulus and did not lead to systemic induction in roots. Interestingly, defoliation by both means strongly induced this gene in panicles, which was not reflected by starch accumulation in these tissues. These above results show that the expression of these starch biosynthesis genes in *Ac* plants is tissue-dependent and is influenced by the type of damage used for defoliation. However, the expression patterns did not coincide with the short- and long-term changes in starch levels observed in response to defoliation. This could be reflecting the complex post-translational regulation of starch synthesis known to operate in plants [84-86]. However, the strong and late expression of *AhSS-IV*, observed in leaves of both HD and MD plants at 30 dppd, could have contributed to the recovery of foliar starch levels in defoliated plants, similar to those in intact controls (Table 4). This possibility is supported by the important role played by soluble starch synthase genes in starch biosynthesis in *Arabidopsis*[87,88].

**Figure 7:**
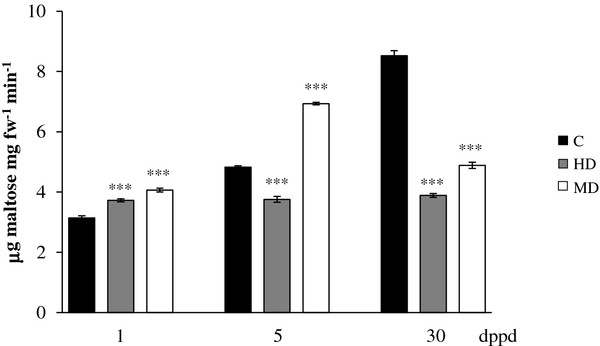
**Amylolytic activity levels measured as μg of maltose per mg of fresh weight released per minute at different days post partial defoliation (dppd) in source leaves of intact control and defoliated *Amaranthus cruentus *plants. ** Defoliation was produced either by insect herbivory (HD) or mechanical damage (MD). For further details refer to Figure 1.

On the other hand, the short-term reduction of the leaf starch reserves which was followed by a subsequent recovery, correlated inversely with the pattern of amylolytic activity shown in Figure 7. Hence, induced activity at 1 and 5 dppd (except in HD plants at 5 dppd), coincided with reduced starch levels, whereas an almost two-fold reduction of activity at 30 dppd might have been associated with the recovery of foliar starch levels in defoliated plants. The expression pattern of a β-amylase-1 gene in leaves of defoliated plants (Table 4) was also consistent with the observed changes in both starch levels and amylolytic activity. These results were in agreement with a report showing that starch breakdown is the primary function of β-amylase in plants [89], and also with several studies in which the starch degradation produced in response to defoliation was found to correlate with augmented amylolytic activity and/or induced amylase gene expression [90-92].

The induced expression of a putative *SnRK1* regulatory gene in leaves of defoliated plants suggests the involvement of this gene in the control of C partitioning produced in response to leaf loss, presumably to alter resource allocation to allow increased tolerance to defoliation, similarly to what was reported in wild tobacco [93]. Interestingly, the expression of this gene was fast and transient in HD plants, whereas it was delayed and persistent in MD plants (Table 4).

### Changes in the expression of a sucrose transporter gene (*AhSUT1*) produced in response to partial defoliation in *A. cruentus*

Even though the mechanism of phloem loading in *Ac* plants is not known, the identification of *AhSUT1*, coding for a sucrose transporter gene, supports the participation of an apoplastic phloem loading process, similarly to what has been reported in several other plant species [94].

This gene was also differentially affected by the way *Ac* plants were defoliated, being more responsive to HD than to MD. The effect was also tissue-, and time-dependent, since *AhSUT1* was strongly induced in HD plants in leaves (at 1 dppd) and panicles, whereas the effect in roots was negative at 5 dppd; roots of MD plants also showed repression of this gene at 30 dppd. In stems, a strong early repression was observed in both HD and MD plants at 1 dppd. In HD, this effect was reversed at 30 dppd (Table 5). A close resemblance with several other studies showing a rapid and transient expression of SUC transporters in response to defoliation by grazing or insect herbivory [21,71,90,95,96], supports an important role for AhSUT1, as a facilitator of SUC transport from the leaves, in the tolerance response to defoliation in grain amaranth.

**Changes in the expression of a putative phosphoenolpyruvate/ Pi translocator** (***AhPPT***) **gene in response to partial defoliation in*A. cruentus*: possible relationship with transient fatty acid accumulation in roots.**

**Figure 8:**
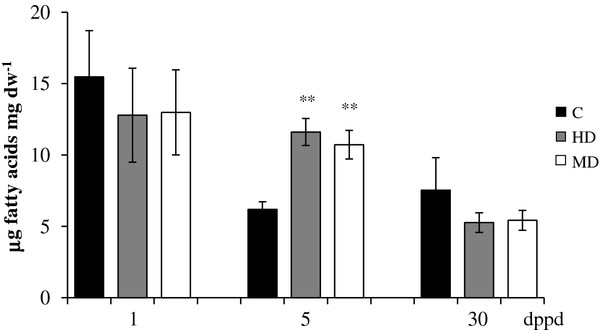
**Total fatty acid levels in μg of fatty acids per mg of dry weight measured at different days post partial defoliation (dppd) in roots of intact control and defoliated *Amaranthus cruentus *plants. ** Defoliation was produced either by insect herbivory (HD) or mechanical damage (MD). For further details refer to Figure 1.

In contrast to most genes examined, the pattern of expression of a putative phosphoenolpyruvate/ Pi translocator (*AhPPT*) showed that, except for a few cases, MD induced a generalized accumulation of *AhPPT* transcripts in all tissues examined, which was particularly strong in roots (Table 5). The strong induction of this gene detected in roots of both HD and MD plants at 5 dppd correlated with the transient accumulation of fatty acids detected in roots (Figure 8; Additional file 10). Such correlation was in agreement with the fact that PPTs are part of a known spectrum of plastidic phosphate translocators in non-photosynthetic plastids that deliver phosphoenolpyruvate (PEP) from the cytosol to plastids to support fatty acid biosynthesis via the action of pyruvate kinase [97,98]. In addition, the high expression this gene in leaves, at 5 dppd, could have been needed to import PEP into chloroplasts to support tissue recovery after damage [99].

**Changes in the expression of a putative Kunitz protease inhibitor (*AhKTI*) and lipoxygenase (*AhLOX2*) genes as markers of the wound response and jasmonic acid signaling in partially defoliated *A. cruentus.***

The protease inhibitor *AhKTI* gene used as a marker of the wound response showed the expected rapid and stable induction in leaves in response to HD and MD, as shown by a significant increase in expression at 1 and 5 dppd (Table 6). The stronger effect on *AhKTI* expression produced by HD was consistent with the differential expression of trypsin inhibitor activity produced in leaves of *A. hypochondriacus* plantlets subjected to mechanical wounding or herbivory [100]. The induction of this gene in unwounded tissues, such as roots of HD (at 1 dppd) and MD (at 5 dppd) plants, and stems of MD plants (which showed a biphasic pattern of expression at 1 and 30 dppd) further supports the generation of a yet undefined wound induced signal needed for the systemic expression of this and most other genes analyzed in this study. Defoliation also led to unexpectedly high levels of *AhKTI* expression in panicles. Also interesting was the delayed down-regulation of *AhKTI* observed at 30 dppd in leaves of MD plants. In contrast, *AtLOX2* used as a marker of JA synthesis, was predominantly expressed in leaves of HD plants, although altered expression of this gene was also detected in roots, where MD led to an early induction at 1 dppd, whereas HD repressed it (Table 6). A clear repression of this gene was detected in stems of both HD and MD plants at 5 dppd. The high expression of the *AhLOX2* gene in leaves of HD plants was somehow expected considering the well-established spike in JA levels that is usually produced when plants are attacked by leaf chewing herbivores in order to induce resistance or tolerance responses [101-103]. Moreover, high *AtLOX2* expression in leaves of HD plants, presumably leading to augmented JA levels, could have been a factor influencing the noticeable difference between gene expression between HD and MD plants observed in this study. Insect herbivory might have also impacted the stress signaling network through its effect on reactive oxygen species (ROS) synthesis and cellular redox metabolism [104]. Likewise, the possible role of JA as a systemic signal for the distal induction of genes in HD plants is in agreement with data generated in *A. hypochondriacus* showing that jasmonates can modulate defense responses vs. insects [8,100] and strongly induce the systemic expression of various herbivory-responsive genes including *AOC*, a JA biosynthetic gene [105]. It is important to notice that JA can also induce changes in resource allocation to enhance plant tolerance to defoliation [101,106-108]. However, the proposed signaling role of JA in resistance/ tolerance responses in grain amaranth remains to be determined.

Different responses to insect herbivory and mechanical damage have been frequently reported in plants and are attributed to the inability to simulate insect herbivory by mechanical means, which often underestimate the damage produced by insect feeding. Moreover, instantaneous removal of leaf tissue by mechanical means is markedly different from the slower tissue removal caused by insect mandibles. Other subtle effects not generally recognized can be caused by trampling, defecating or the dispersal of pathogens by the herbivore [28], whereas a well-defined determinant of response differentiation is the insect’s saliva. In this respect, it has been amply demonstrated that resistance signaling is elicited differently from simple wounding when herbivore-specific elicitors (e.g. fatty acid conjugates such as volicitin) contained in the insect’s saliva or oral secretions are introduced into wounds [109-112].

### Changes in the expression of a senescence associated (*AhSAG*) gene as a marker of ageing and development in partially defoliated in *A. cruentus*

The expression of the *AhSAG* gene, used as a marker of development and senescence, produced the expected pattern in leaves of HD plants, where a reduction/repression of its expression at 1 dppd was followed by clearly augmented levels at 5 and 30 dppd (Table 6). A similar tendency, although weaker, was observed in leaves and stems MD plants, whereas in stems of HD plants, the expression of the *AhSAG* gene was strongly repressed. The early expression in roots of HD and MD plants at 1 dppd suggests that this gene may be a systemic marker of wound stress and not development, in this tissue. This is supported by several reports showing that several genes that are up-regulated during senescence also accumulate in response to biotic and abiotic stress [113]. Panicles of HD plants showed a very high expression of this gene. This was probably reflecting the accelerated onset of the flowering stage consistently observed in HD plants (results not shown). The differential effect of HD and MD on gene expression was again evidenced in stems, where a clearly contrasting effect on the expression of *AhSAG* was observed at 30 dppd.

In addition to early flowering, HD reduced leaf longevity and increased branching (data not shown). These changes, however, did not negatively affect plant height or seed yield in physiologically mature plants (data not shown). These changes indicate that insect herbivory produced a stronger ontogenetic shift than mechanical wounding in grain amaranth. The difference was manifested by premature flowering (an escape strategy?) and the accelerated senescence of mature leaves. This is in partial agreement with a related study that found that clipping in *A. cruentus* produced drastic changes in plant architecture, and led to an overcompensation response that increased seed yield [17].

## Conclusions

Grain amaranth plants are known to thrive under harsh ambient conditions that are inhospitable to other crops, particularly cereals. The mechanisms responsible for drought and saline stress resistance have been attributed to several physiological, biochemical and/ or genetic adaptations, including superior water use efficiency, changes in root length and architecture, the accumulation of compatible solutes and/ or the expression of key stress-related genes [30]. Conversely, not much is known regarding the observed ability of grain amaranth to sustain defoliation. The NSC data obtained in this work showed that a single defoliation event in young grain amaranth plantlets led to a rapid reduction in NSC levels in both sink tissues and source leaves that recovered some time after defoliation. We propose that the rapid mobilization of foliar starch reserves followed by an efficient recovery of all NSC reserves supported the tolerance response to defoliation in grain amaranth, as manifested by unaltered plant growth and reproductive fitness. Moreover, the long-term sequestration of starch in roots and stems that was observed when the starch levels in leaves were unusually high, together with the temporary accumulation of fatty acids in roots, suggest a plastic response to defoliation in grain amaranth. Remarkably, only a few changes in gene expression and enzyme activity could be associated with the above changes. This outcome was in agreement with the highly complex nature of the CHO metabolic flux in plants, over which individual genes are predicted to have only a limited influence, if any. Also large differences in gene expression patterns were observed between HD and MD plants, even though the responses of plants to both treatments were very similar. This could have been associated with the stronger ontogenetic shift caused insect herbivory, which was manifested by premature flowering, altered plant architecture and accelerated leaf senescence.

In accordance with this and other related studies, it can be concluded that a better understanding of how C partitioning influences tolerance to defoliation, and perhaps to other stresses including excessive salinity [36], has a great potential for use in the future improvement of cultivated crops, particularly those that are stress and defoliation intolerant, such as maize.

## Methods

### Plant material, insects and treatments

Seeds of the two genotypes employed in this study, *Amaranthus cruentus* cultivar “Tarasca” and *Amaranthus hypochondriacus* cultivar “Revancha”, were kindly provided by Eduardo Espitia (INIFAP, México). Seeds were germinated as described previously [30]. Larvae of the Hawaiian beet webworm *Spoladea recurvalis*, a common pest of amaranth and related species, were reared in a colony established from specimens collected in nearby fields. The seedlings were subsequently transplanted to 16 L plastic pots, containing sterile general soil mixture, 21 days after germination, and were transported to a greenhouse where the experiments were conducted. The plantlets were fertilized once, one week after transplant, with a 20: 10: 20 (N: P: K) nutrient soil drench solution according to the manufacturer’s instructions (Peters Professional; Scotts-Sierra Horticultural Products, Marysville, OH, USA) until they had 6 to 8 expanded leaves. All herbivory experiments were performed with 30-days-old *A. cruentus* plants in a greenhouse localized at Cinvestav-Irapuato, México, (20°40^′^18^″^N 101°20^′^48^″^W) under natural conditions of light and temperature. The choice of *A. cruentus* for the defoliation experiments was based on its insensitivity to the photo-period, a useful characteristic which allowed extended experimentation during early and late periods of the year, which are unsuitable for *A. hypochondriacus*[12]. Defoliation by insect herbivory was performed by placing three larvae per plant for 4 days, which resulted in a leaf tissue loss of approximately 30%. Defoliation by mechanical damage was performed by removing the same percentage of foliar tissue with a 0.5 cm diameter cork-borer. Care was exercised to match the pattern of mechanical tissue removal with that of herbivory. Samples of damaged source leaves (three per plant), stem (15 cm segments, starting from the base) and roots were collected from three plants at 0, 1, 5 and 30 days after the defoliation treatment. Panicles were sampled soon after their emergence, 30 days after treatment. Control experiments with undamaged plants were performed simultaneously. Plant height and total number of leaves were measured at the three time points. The number of internodes per stem was determined at 30 dppd. In two experiments, seeds were harvested from groups of control and defoliated plants allowed to reach full maturity (≥ 110 dppd). Tissue samples of each three plant group were pooled and were flash frozen with liquid N_2_ and stored at −80°C until use. Pooled samples were used for analysis of NSC, fatty acids, gene expression and sucrolytic activity levels. Each experiment was repeated three times, once in the fall-winter of 2010 and twice in the spring of 2011.

Leaf tissue of intact *A. hypochondriacus* and *A. cruentus* plants was used for RNA extraction and genomic DNA extraction for full-length cDNA and gene isolation.

### Extraction of total RNA and cDNA preparation

Total RNA was extracted from 100–200 mg of frozen tissue with the Trizol reagent (Invitrogen, Carlsbad, CA, USA), according to the manufacturer’s instructions, with modifications. These consisted of the addition of a salt solution (sodium citrate 0.8 M + 1.2 M NaCl) during precipitation in a 1:1 v/v ratio with isopropanol and further purification with LiCl (8 M) for one hour at 4°C. All RNA samples were analyzed by formaldehyde agarose gel electrophoresis and visual inspection of the ribosomal RNA bands upon ethidium bromide staining. Total RNA samples (1 μg) were reverse-transcribed to generate the first-strand cDNA using an oligo dT_20_ primer and 200 units of SuperScript II reverse transcriptase (Invitrogen).

### Gene expression analysis by quantitative real-time RT-PCR (qRT-PCR)

The cDNA employed for the qRT-PCR assays was initially prepared from 4 μg total RNA. It was then diluted ten-fold in sterile deionized-distilled (dd) water prior to qRT-PCR. Amplifications were performed using SYBR Green detection chemistry and run in triplicate in 48-well reaction plates with the StepOne™ Real-Time PCR System (Applied Biosystems, Perkin-Elmer, Foster City, CA, USA). Reactions were prepared in a total volume of 8 μl containing: 1 μl of template, 0.8 μl of each amplification primer (2 μM), 4 μl of SYBR® Green JumpStart™ *Taq* Ready Mix™ (Sigma-Aldrich St. Louis, MO, USA) and 1.4 μl of sterile dd water. Quantitative real-time PCR was performed in triplicate for each sample using the primers listed in Additional file 11. Primers were designed for each gene, based on partial cDNA sequences derived from the transcriptomic analysis of *Ah*[30] or from complete cDNAs generated in this study (see above). Primer design was performed using DNA calculator software (Sigma-Aldrich) and included, when possible, part of unique 3’ non-coding regions to ensure specificity.

The following protocol was followed for all qRT-PCR runs: 15 min at 95°C to activate the JumpStart™ *Taq* Polymerase (Sigma-Aldrich), followed by 40 cycles of denaturation at 95°C for 15 s and annealing at 60°C for 1 min. Slow amplifications requiring an excess of 32 cycles were not considered for analysis. The specificity of the amplicons was verified by melting curve analysis after 40 cycles and agarose gel electrophoresis. Baseline and threshold cycles (Ct) were automatically determined using Real-Time PCR System software. PCR efficiencies for all genes tested were greater than 95%. Relative expression was calculated using the comparative cycle threshold method [114], where delta (Δ) cycle threshold of cDNA from undamaged controls was defined as 100% transcript presence.

Transcript abundance data were normalized against the average transcript abundance of three reference genes: *actin* (isotig 10321), *β-tubulin* (isotig 05486) and *elongation factor 1α* (*EF1α*) (isotig 13098). These were obtained from the above transcriptomic study. The fold change in expression of the target genes in each treatment was calculated using the following equation: 2^-ΔΔCt^, where ΔΔCt = (Ct target gene - average Ct reference genes)_treatment_ - (Ct target gene - average Ct reference genes)_control_. Values reported are the mean of three repetitions ± SE of one representative experiment. The qRT-PCR expression analysis of the majority of the genes included in this study, except for all sucrolytic, *AGP* and invertase inhibitor genes that were previously shown to have reproducible patterns of expression by semi-quantitative RT-PCR assays (not shown), was validated in at least two independent experiments.

### Full-length cDNA amplification

In order to amplify full-length *AhSuS-1*, *AhSuS-2*, *AhAGPS-1*, *AhA/NI-1* and *AhVI-1* cDNAs, total RNA samples (1 μg) from leaves of grain amaranth plantlets were reverse-transcribed to generate the first-strand cDNA as described above. An aliquot of these reactions (2 μl) was then directly used as template in all PCR reactions in the presence of 100 pmol each of specific primers designed on the basis of sequences obtained from the *Ah* transcriptome [30] (see Additional file 12). The fragments obtained were cloned and sequenced to confirm that they corresponded to the gene of interest. The amplification of the 5’ and 3’ cDNA ends was carried out by RACE (Rapid Amplification of cDNA Ends) with the SMARTer™ RACE cDNA Amplification Kit (Clontech, Laboratories, Mountain View, CA), according to the manufacturer’s instructions. All complete cDNA sequences were deposited in the GenBank as JQ012918 (*AhSuS-1*), JQ012919 (*AhSuS-2*), JQ012920 (*AhA/NI-1*), JQ012921 (*AhVI-1*) and HM021763 (*AhAGPS-1*).

### PCR amplification of a partial cDNA sequences of cell wall invertase (AhCWI)

Degenerate oligonucleotides OIN3 (5^′^ CCTTCACYTNTTYTAYCARYAYAAYCC 3^′^, [115] and INV5 (5^′^ NGTCTTGGWDGCGTAAATAYTTMCCATA 3^′^) were deduced from conserved cell wall invertase (*CWI*) sequences in *Beta vulgaris* (AJ278531)*, Chenopodium rubrum* (X81792-94) and *Daucus carota* (M58362.1), and used as primers for PCR amplification. PCR reactions were performed in a 25 μl reaction volume with 2 μl cDNA from *A. hypochondriacus* as template, primers (100 pmol each), dNTPs (100 mM each), 10 × Taq reaction buffer, 50 mM MgCl_2_ and 1 U of *Taq* polymerase (Invitrogen). After a pre-denaturing step at 94°C for 3 min, the amplification consisted of 35 cycles of 30 s at 94°C, 30 s at 60°C and 45 s at 72°C and a final extension of 10 min at 72°C. The resulting PCR fragments were separated and purified by agarose gel electrophoresis. The PCR fragments from the major bands were purified, cloned and sequenced.

### Full length genomic amplification

Genomic DNA was extracted from leaves of *A. hypochondriacus* plantlets as instructed [116] and digested with four different restriction enzymes (*DraI*, *EcoRV*, *PvuI*, *StuI*). The resulting fragments were blunt-end ligated to the Genome-Walker Adaptor provided by the Genome-Walker kit (Clontech) to generate the corresponding libraries. These libraries were used as templates for PCR and nested PCR using primers (see Additional file 12) designed on the complete cDNAs for *AhVI-1* and *AhAGPS-1* obtained previously (see above). Amplifications were done in both 3' and 5' directions to obtain the complete sequences of these genes, including a sizeable portion of their promoter regions. The overlap between the genomic sequences thus obtained and their respective cDNA templates confirmed the identity of the newly generated fragments. All PCR amplicons obtained were cloned using the TOPO TA cloning kit (Invitrogen) and sequenced. Both *AhVI-1* and *AhAGPS-1* genomic sequences were deposited in the GenBank as JQ012921 and JQ034321, respectively.

### DNA sequencing and sequences analysis

Recombinant plasmid DNA was prepared and then sequenced. Sequencing was provided as a service by the National Laboratory of Genomics for Biodiversity (Langebio, at Cinvestav- Irapuato) and the Biotechnology Institute (IBT-UNAM, México). Computer analysis was performed using FastPCR 6.0, AnnHyb 4.944 and Chromas-Lite 2.01 software. Sequence homologies were verified against GenBank databases using BLAST programs [117].

### Phylogenetic analyses

Alignment of amino acid sequences and phylogenetic analyses were conducted using the PhyML method within the Bosque 1.7.157 software.

### Putative sub-cellular localization

Putative sub-cellular localization of the genes was performed *in silico* by the following programs, available online: PSORT, SignalP, TargetP, Protein Prowler and MitoProt. (psort.hgc.jp/form.html, http://www.cbs.dtu.dk/services/TargetP/, pprowler.imb.uq.edu.au/, http://www.cbs.dtu.dk/services/SignalP/ and ihg.gsf.de/ihg/mitoprot.html).

### Bio-informatic analysis

The promoter sequences of the *AhVI-1* and *AhAGPS-1* genes were subjected to an *in silico* analysis with the following databases: PLACE (http://www.dna.affrc.go.jp/PLACE/), PlantCARE (http://bioinformatics.SlPSb.ugent.be/wetools/ plantcare/html/), the SolGenomics Network (solgenomics.net) and the Genomatix software suite (http://www.genomatix.de) in order to identify the presence of putative cis-regulatory elements.

### Determination of non-structural carbohydrate levels

All tissues (leaves, stems, roots and panicles) were collected at the beginning of the dark period (6:30 p.m.) and flash frozen in liquid nitrogen. Frozen ground tissue (200 mg) was extracted with 500 μl 80% aqueous ethanol (v/v) and incubated at 4°C for 10 min with stirring. After refrigerated centrifugation at 10,000 rpm (4°C for 10 min), the cleared supernatants were transferred into new tubes and concentrated by centrifugation (Heto Maxi Dry Lyo, Heto-Holten, Denmark). The residue was re-dissolved in 500 μl of 100 mM Hepes buffer, pH 7.4, and 5 mM MgCl_2_, and used for the determination de soluble sugars. The pellet derived from the centrifugation step was used for the determination of starch. To this end, it was homogenized with 500 μl of 10 mM KOH and incubated at 99°C for two hours. Sucrose (SUC), glucose (GLC), fructose (FRC) and starch contents were measured using enzyme-based methods as instructed (Boehringer Mannheim/R-Biopharm, Darmstadt, Germany), except that the final reaction volume was reduced to fit a micro-plate format (250 μl per reaction).

### Determination of fatty acid levels

Fatty acids were determined according to [118] with modifications: i) the methylation step was performed with BF_3_ at slightly harsher conditions (100°C for 15 min), and ii) the organic phase was dried under a stream of N_2_ and re-dissolved in isooctane before loading into the gas chromatograph.

### Invertase, sucrose synthase and amylase activities in vitro

Acid soluble (vacuolar) and insoluble (cell wall), neutral (cytoplasmic) invertase and sucrose synthase activities were determined according to [119,120]. Total amylolytic activity was determined as described in [121].

### Statistical analysis

The statistical analysis of the data was performed in the R statistical language (R Development Core Team 2004, Version 1.9.0; http://www.R-project.org).

## Competing interests

The authors declare that they have no competing interests.

## Authors’ contributions

JPDF designed and coordinated the study. PACA performed all the experiments. She was aided by HAA in statistics and genomics and by NAMG in the metabolite and enzymatic assays. PACA and JPDF analyzed the data. ATF helped with the Real-Time PCR method. JPDF and ATF wrote the manuscript. All authors read and approved the final version of the manuscript.

## Supplementary Material

Additional file 1 Characteristics of the grain amaranth genes selected for analysis in different tissues of defoliated plants to determine their possible role in C mobilization and tolerance. Click here for file

Additional file 2 Comparison of deduced amino acid sequences of plant sucrose synthases. Click here for file

Additional file 3 Comparison of deduced amino acid sequences of plant invertases. Click here for file

Additional file 4 Comparison of deduced amino acid sequences of plant ADP-glucose pyrophosphorylases (AGP). Click here for file

Additional file 5 **Phylogenetic dendogram of known plant invertase inhibitors.** (DOCX 77 kb) Click here for file

Additional file 6 **Structure of grain amaranth genes coding for a small subunit of ADP glucose pyrophosphorylase (*AhAGPS-1*) and a vacuolar invertase (*AhVI-1*).** Click here for file

Additional file 7 **Regulatory elements identified in the promoter regions of the *AhAGPS-1 *and *AhVI-1 *genes. The 5’ regulatory region was analyzed using PLACE, PlantCARE and Genomatix Matinspector databases.** Click here for file

Additional file 8 **Comparison of the 5’ regulatory regions identified in the ADP-glucose pyrophosphorylase and vacuolar invertase genes of *Solanum tuberosum, S. lycopersicum, Beta vulgaris *and *Amaranthus hypochondriacus. *** Click here for file

Additional file 9 Results of an atypical experiment showing a long-term accumulation of starch in stems and roots of MD plants. Click here for file

Additional file 10 Fatty acid composition in roots of control and defoliated grain amaranth plants. Click here for file

Additional file 11 Primers used for gene expression analysis by qRT PCR. Click here for file

Additional file 12 **Primers used to amplify the 5 'and 3' cDNA ends (RACE) and the genomic sequences, including part of the promoter regions, of the *AhVI-1 *and *AhAGPS-1 *genes. ** Click here for file
