# Supplementary material for: Metabolic and enzymatic changes associated with carbon mobilization, utilization and replenishment triggered in grain amaranth (Amaranthus cruentus) in response to partial defoliation by mechanical injury or insect herbivory
Source: BMC Plant Biol. 2012 Sep 12;12:163. doi: 10.1186/1471-2229-12-163 (PMC3515461; doi:10.1186/1471-2229-12-163)
Supplement: Additional file 1 — Characteristics of the grain amaranth genes selected for analysis in different tissues of defoliated plants to determine their possible role in C mobilization and tolerance. [file 1471-2229-12-163-S1.doc]

**Additional file 1. Characteristics of the grain amaranth genes selected for analysis in different tissues of defoliated plants to determine their possible role in C mobilization and tolerance.**

| **Gene** | **No. isoforms analyzed** | **Criteria for selection** | **Refs.** |
| --- | --- | --- | --- |
| ***AhVI-1*** | 1 | Preliminary analysis in amaranth plants revealed that this isoform is regulated by development and induced by insect herbivory. Known to be necessary to maintain cell growth in sink tissues when CHO in source leaves are depleted. | 1 |
| ***AhCWI*** | 1 | Only CWI isoform detected in *Ah*’s transcriptome. | 2 |
| ***AhA/NI*** | 2 | Both isoforms are localized to the chloroplast, which is important in the regulation of carbon partitioning between the cytosol and chloroplasts. Induced by several types of stress. |  |
| ***AhSuS*** | 2 | *AhSuS-1* and *AhSuS-2* were differentially expressed by stress conditions in the RNA-seq analysis of *Ah* transcriptomic data. They are highly homologous to *B. vulgaris’* isoforms induced by stress. *SuS* genes are known to determine starch levels in potato tubers and Arabidopsis seeds. | [2, 6-10] |
| ***AhInvI*** | 3 | Preliminary analysis in amaranth plants showed that their expression showed an inverse correlation with the expression of vacuolar and apoplastic acid invertases. | 11 |
| ***AhγVPE*** | 1 | Found to be differentially expressed in the RNA-seq analysis of *Ah* transcriptomic data. Has a predicted role in the control of vacuolar invertase activity. | [2, 12-13] |
| ***AhAGPS*** | 1 | Only small subunit isoform detected in *Ah*’s transcriptome. Key involvement in starch synthesis. | [2, 14] |
| ***AhAGPL*** | 2 | Both isoforms were found to be differentially expressed by stress conditions in the RNA-seq analysis of *Ah* transcriptomic data. Highly homologous to stress-induced isoforms in tomato. Key involvement in starch synthesis. | [2, 14] |
| ***AhSSIII*** | 1 | Only isoform detected in *Ah*’s transcriptome. Similar to an Arabidopsis’ isoform known to play a key role in starch accumulation. | [2, 15] |
| ***AhSSIV*** | 1 | Same as above. | [2, 15] |
| ***AhGBSS*** | 1 | Only isoform detected in *Ah*’s transcriptome. Differentially expressed by stress conditions in the RNA-seq analysis of *Ah* transcriptomic data. Responsible for amylose content in starch. | [2, 16] |
| ***AhPPT*** | 1 | Differentially expressed by stress conditions in the the RNA-seq analysis of *Ah* transcriptomic data. Transports phosphoenolpyruvate from the cytosol to the choloroplast and plastids of non-photosynthetic tissues for the biosynthesis of fatty acids and other metabolites. | [2, 17] |
| ***AhG6PT*** | 1 | Differentially expressed by stress conditions in the RNA-seq analysis of *Ah* transcriptomic data. High homology with an isoform involved in the microbial volatile induced hyper-accumulation of starch. Also required in fatty acids biosynthesis. | [2, 18-19] |
| ***AhSUT*** | 1 | Differentially expressed by stress conditions in the RNA-seq analysis of *Ah* transcriptomic data. Similar to those reported to facilitate apoplastic phloem loading in other plant species. | [2, 20] |
| ***AhSPS*** | 1 | Differentially expressed by insect herbivory in the RNA-seq analysis of *Ah* transcriptomic data. Key regulator of sucrose synthesis in plants. | [2, 21] |
| ***AhBMY1*** | 1 | Differentially expressed by stress conditions in the RNA-seq analysis of *Ah* transcriptomic data. Similar to the gene encoding for BMY1 in Arabidopsis, an enzyme that accounts for more than 90% of total β-amylase activity in its mesophyll cells. | [2, 22] |
| ***AhSnRK1*** | 1 | The most complete isoform detected in *Ah*’s transcriptome. Has homology with similar serine/threonine protein kinases that regulate the expression of carbon metabolism genes in response to carbon availability | [23 - 26] |
| ***AhLOX2*** | 1 | Marker of jasmonic acid-related responses | [27-29] |
| ***AhKTI*** | 1 | Marker of wounding. Strong accumulation of proteinase inhibitors occurs in grain amaranth subjected to wounding and insect herbivory. Also, several KTI genes are strongly induced in other plants after wounding, herbivory and/ or abiotic stress [43, 44]. | [30-32] |
| ***AhSAG*** | 1 | Marker of developmental and senescence processes. Similar to the *AtSAG18* gene, isolated from mid-senescent leaves of Arabidopsis and in senescent leaves of Arabidopsis plants exposed to ozone. | [33, 34] |

1. Nguyen-Quoc B, Foyer CH: **A role for ‘futile cycles’ involving invertase and sucrose synthase in sucrose metabolism of tomato fruit**. *J Exp Bot* 2001, **52:** 881-889.
2. Délano-Frier JP, Avilés-Arnaut H, Casarrubias-Castillo K, Casique-Arroyo G, Castrillón-Arbeláez PA, Herrera-Estrella L, Massange-Sánchez J, Martínez-Gallardo NA, Parra-Cota FI, Vargas-Ortiz, Estrada-Hernández MG: **Transcriptomic analysis of grain amaranth (*Amaranthus hypochondriacus*) using 454 pyrosequencing: comparison with A. tuberculatus, expression profiling in stems and in response to biotic and abiotic stress**. BMC Genomics 2011, **12:** 363.
3. Xiang L, Le Roy K, Bolouri-Moghaddam MR, Vanhaecke M, Lammens W, Rolland F, Van den Ende W: **Exploring the neutral invertase-oxidative stress defence connection in *Arabidopsis thaliana***. *J Exp Bot* 2011, **62:** 3849-3862.
4. Vargas WA, Pontis HG, Salerno GL: **Differential expression of alkaline and neutral invertases in response to environmental stresses: characterization of an alkaline isoform as a stress-response enzyme in wheat leaves**. *Planta* 2007, 226:1535-1545.
5. Vargas WA, Pontis HG, Salerno GL: **New insights on sucrose metabolism: evidence for an active A/N-Inv in chloroplasts uncovers a novel component of the intracellular carbon trafficking**. *Planta* 2008, **227:** 795-807.
6. Zrenner R, Salanoubat M, Willmitzer L, Sonnewald U: **Evidence of the crucial role of sucrose synthase for sink strength using transgenic potato plants (*Solanum tuberosum* L.)**. *Plant J* 1995, **7:** 97-107.
7. Angeles-Núñez JG, Tiessen A: **Arabidopsis sucrose synthase 2 and 3 modulate metabolic homeostasis and direct carbon towards starch synthesis in developing seeds**. *Planta* 2010**, 232:**701-718.
8. Haagenson DM, Klotz KL, McGrath, JM: **Sugarbeet sucrose synthase genes differ in organ-specific and developmental expression**. *J Plant Physiol* 2006, **163:** 102-106.
9. Hesse H, Willmitzer L: **Expression analysis of a sucrose synthase gene from sugarbeet (*Beta vulgaris* L.)**. *Plant Mol Biol* 1996, **30:** 863-872.
10. Klotz KL, Haagenson DM: **Wounding, anoxia and cold induce sugarbeet sucrose synthase transcriptional changes that are unrelated to protein expression and activity***. J Plant Physiol* 2008, **165:** 423-434.
11. Castrillón-Arbeláez PA, Délano-Frier JP: **The sweet side of inhibition: invertase inhibitors and their importance in plant development and stress responses.** *Curr Enz Inhib* 2011, **7:** 169-177.
12. Koch K: **Sucrose metabolism: regulatory mechanisms and pivotal roles in sugar sensing and plant development**. *Curr Opin Plant Biol* 2004, **7:** 235-246.
13. Yamada K, Shimada T, Nishimura M, Hara-Nishimura I: **A VPE family supporting various vacuolar functions in plants**. *Physiol Plant* 2005, **123:** 369-375.
14. Yin Y Kobayashi Y, Sanuki A, Kondo S, Fukuda N, Ezura H, Sugaya S, Matsukura C: **Salinity induces carbohydrate accumulation and sugar regulated starch biosynthetic genes in tomato (*Solanum lycopersicum* L. cv. ‘Micro-Tom’) fruits in an ABA- and osmotic stress-independent manne**r. *J Exp Bot* 2010, **61:** 563-574.
15. Szydlowski N, Ragel P, Raynaud S, Roldán I, Montero M, Lucas MM, Roldán I, Montero M, Muñoz FJ, Ovecka M, Bahaji A, Planchot V, Pozueta-Romero J, D’Hulst C, Mérida A: **Starch granule initiation in *Arabidopsis* requires the presence of either class IV or class III starch synthase**. *Plant Cell* 2009, **21:** 2443-2457.
16. Lindeboom N, Chang PR, Tyler RT, Chibba RN: **Granule-Bound Starch Synthase I (GBSSI) in Quinoa (*Chenopodium quinoa* Willd.) and its relationship to amylose content**. *Cereal Chem* 2005, **82:** 246-250.
17. Prabhakar V, Löttgert T, Geimer S, Dörmann P, Krüger S, Vijayakumar V, Schreiber L, Göbel C, Feussner K, Feussner I, Marin K, Staehr P, Bell K, Flügge UI, Häusler RE: **Phosphoenolpyruvate provision to plastids is essential for gametophyte and sporophyte development in *Arabidopsis thaliana***. *Plant Cell* 2010, **22:** 2594-2617.
18. Zhang X, Szydlowski N, Delvalle, D, D’Hulst C, James MG, Myers AM: **Overlapping functions of the starch synthases SSII and SSIII in amylopectin biosynthesis in *Arabidopsis***. *BMC Plant Biol* 2008, **8:** 96.
19. Li J, Ezquer E, Bahaji A, Montero M, Ovecka M, Baroja-Fernández E, Muñoz FJ, Mérida A, Almagro G, Hidalgo M, Sesma MT, Pozueta-Romero J: **Microbial volatile-induced accumulation of exceptionally high levels of starch in *Arabidopsis* leaves is a process involving NTRC and Starch Synthase classes III and IV**. *Mol Plant-Microbe Interact* 2011, **24:** 1165-1178.
20. Sauer N: Molecular physiology of higher plant sucrose transporters. *FEBS Lett* 2007 581: 2309-2317.
21. Stitt M, Wilke I, Feil R, Heldt HW: **Coarse control of sucrose phosphate synthase in leaves: alterations of the kinetic properties in response to the rate of photosynthesis and the accumulation of sucrose**. *Planta* 1988, **174:** 217-230.
22. Monroe JD, Preiss J: **Purification of a β-amylase that accumulates in *Arabidopsis thaliana* mutants defective in starch metabolism**. *Plant Physiol* 1990, **94:** 1033-1039.
23. Tiessen A, Hendriks JHM, Stitt M, Branscheid A, Gibon Y, Farré EM, Geigenberger P: **Starch synthesis in potato tubers is regulated by post-translational redox modification of ADP-glucose pyrophosphorylase: a noble regulatory mechanism linking starch synthesis to the sucrose supply**. *Plant Cell* 2002, **14:** 2191-2213.
24. Tiessen A, Prescha K, Branscheid A, Palacios N, McKibbin R, Halford NG, Geigenberger P: **Evidence that SNF1- related kinase and hexokinase are involved in separate sugar signaling pathways modulating post-translational redox activation of ADP-glucose pyrophosphorylase in potato tubers**. *Plant J* 2003, **35:** 490-500.
25. Halford NG, Hardie DG: **SNF1-related protein kinases: global regulators of carbon metabolism in plants?** *Plant Mol Biol* 1998, **37:** 735-748.
26. Halford NG, Paul MJ: **Carbon metabolite sensing and signalling**. *Plant Biotechnol J* 2003, **1:** 381-398.
27. Baldwin IT: **Methyl jasmonate-induced nicotine production in *Nicotiana attenuata*: inducing defenses in the field without wounding**. *Entomol Exp Appl* 1996, **80:** 213-220.
28. Thaler JS, Stout MJ, Karban R, Duffey SS: **Exogenous jasmonates simulate insect wounding in tomato plants (*Lycopersicon esculentum*) in the laboratory and field**. *J Chem Ecol* 1996, **22:** 1767-1781.
29. Babst BA, Ferrieri RA, Gray DW, Lerdau M, Schlyer DJ, Schueller M, Thorpe MR, Orians CM**: Jasmonic acid induces rapid changes in carbon transport and partitioning in *Populus***. *New Phytol* 2005, **167:** 63-72.
30. Sánchez-Hernández C, Martínez-Gallardo N, Guerrero-Rangel A, Valdés-Rodríguez S, Délano-Frier J: **Trypsin and a-amylase inhibitors are differentially induced in leaves of amaranth (*Amaranthus hypochondriacus*) in response to biotic and abiotic stress**. *Physiol Plant* 2004 **122:** 254-264.
31. Major IT, Constabel CP: **Molecular analysis of poplar defense against herbivory: comparison of wound- and insect elicitor-induced gene expression**. *New Phytol* 2006, **172:** 617-635.
32. Huang H, Qi SD, Qi F, Wu CA, Yang GD, Zheng CC: ***NtKTI1*, a Kunitz trypsin inhibitor with antifungal activity from *Nicotiana tabacum*, plays an important role in tobacco’s defense response**. *FEBS J* 2010, **19:** 4076-4088.
33. Weaver LM, Gan S, Quirino B, Amasino RM: **A comparison of the expression patterns of several senescence associated genes in response to stress and hormone treatment**. *Plant Mol Biol* 1998, **37:** 455-469.
34. Miller JD, Arteca RN, Pell EJ: Senescence-associated gene expression during ozone-induced leaf senescence in Arabidopsis. *Plant Physiol* 1999, 120: 1015-1023.
