# Supplementary material for: Metabolic and enzymatic changes associated with carbon mobilization, utilization and replenishment triggered in grain amaranth (Amaranthus cruentus) in response to partial defoliation by mechanical injury or insect herbivory
Source: BMC Plant Biol. 2012 Sep 12;12:163. doi: 10.1186/1471-2229-12-163 (PMC3515461; doi:10.1186/1471-2229-12-163)
Supplement: Additional file 2 — Comparison of deduced amino acid sequences of plant sucrose synthases. [file 1471-2229-12-163-S2.doc]

**
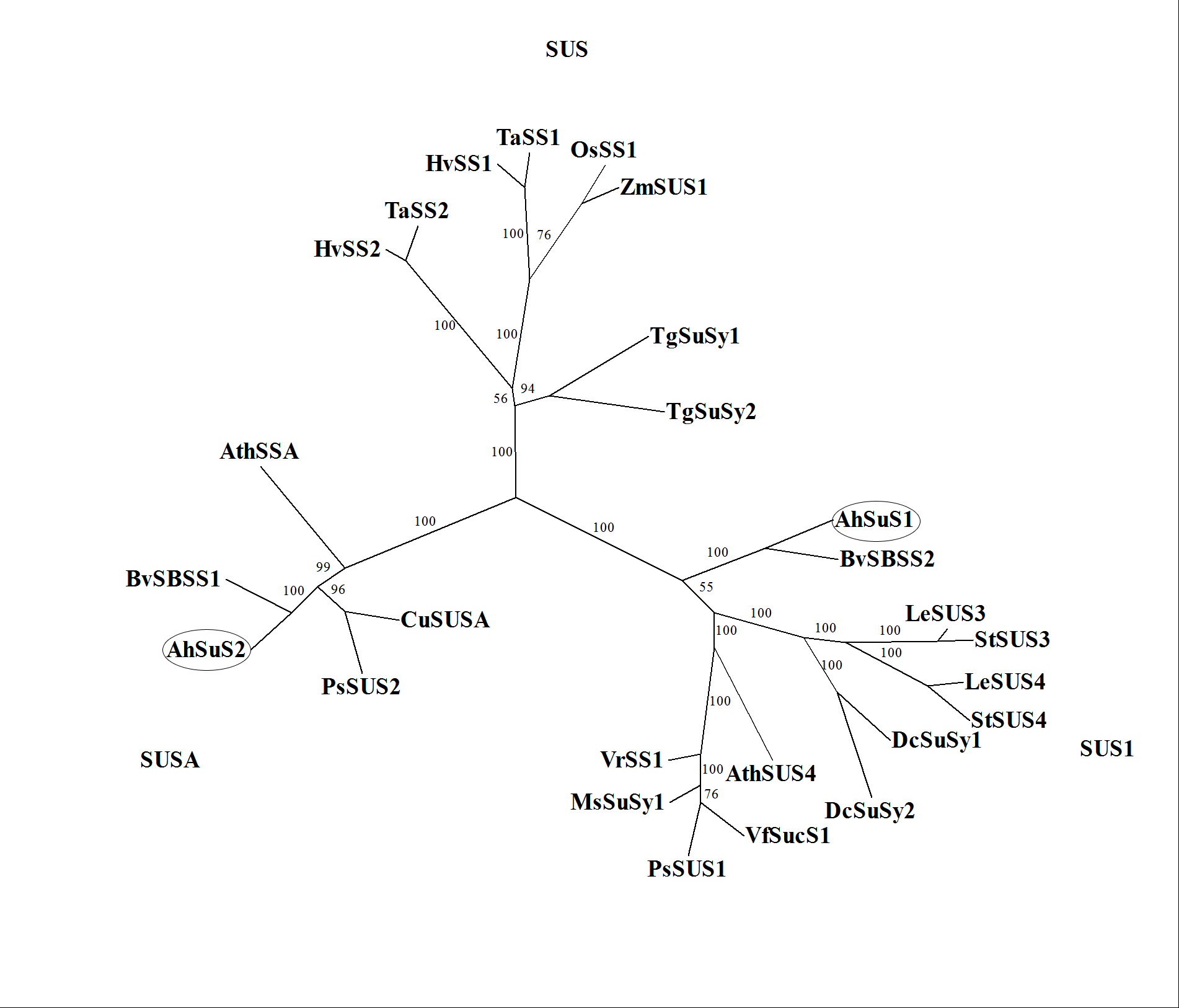
**

**Additional File 2.** Comparison of deduced amino acid sequences of plant sucrose synthases. The SUS, SUSA and SUS1 types were discriminated according to Komatsu et. al. 2002, J Exp Bot 53: 61-71. Amaranth sucrose synthases are encircled. The amino acid sequences were obtained from the following sources, with their respective accession numbers enclosed in parentheses: *Beta* vulgaris, SBSS and SBSS1 (AY457173 and EF660856); *Solanum tuberosum*, StSUS3 and StSUS4 (U24087, U24088); *Lycopersicum esculentum,* LeSUS3 and LeSUS4 (AJ011319, L19762); *Daucus carota,* DcSuSy1 and DcSuSy2 (X75332 and Y16091); *Arabidopsis thaliana,* AthSSA and AthSUS4 (X60987, NM_114187); *Vicia faba,* VfSucS1 (X69773); *Pisum sativum,* PsSUS1 and PsSUS2 (AJ12080, AJ001071); *Medicago sativa,* MsSuSy1 (AF049487); *Vigna radiata*, Vr-SS1 (D10266); *Tulipa gesneriana*, TgSuSy1 and TgSuSy2 (X96938, X96939); *Oryza sativa*, OsSS1 (X64770); Zea mays, ZmSUS1 (X02400); *Triticum aestivum*, TaSS1 and TaSS2 (AJ001117, AJ000153); *Hordeum vulgare*, HvSS1 and HvSS2 (X65871, X69931); *Citrus unshiu*, CuSUSA (AB022091), and ***Amaranthus hypochondriacus* AhSuS-1 and AhSuS-2 (JQ012918, JQ012919).**
