# Supplementary material for: Metabolic and enzymatic changes associated with carbon mobilization, utilization and replenishment triggered in grain amaranth (Amaranthus cruentus) in response to partial defoliation by mechanical injury or insect herbivory
Source: BMC Plant Biol. 2012 Sep 12;12:163. doi: 10.1186/1471-2229-12-163 (PMC3515461; doi:10.1186/1471-2229-12-163)
Supplement: Additional file 3 — Comparison of deduced amino acid sequences of plant invertases. [file 1471-2229-12-163-S3.docx]

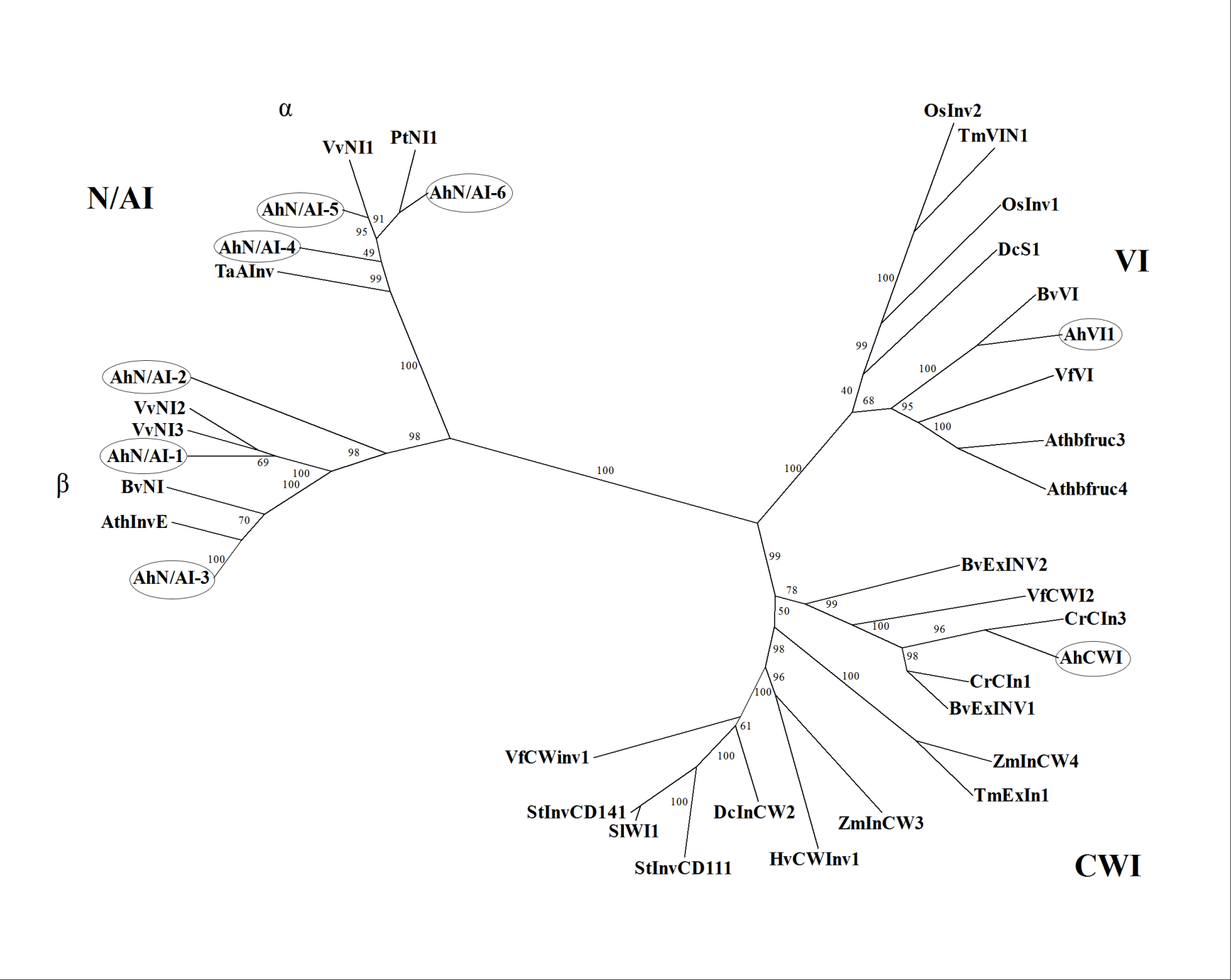


**Additional File 3.** Comparison of deduced amino acid sequences of plant invertases. These sequences are grouped into vacuolar (VI), cell wall (CWI) and neutral/ alkaline (N/ AI) invertases. The last group was divided according to their subcellular localization, either as chloroplastic (β) or cytosolic (α). The invertases studied in this manuscript, are encircled. The deduced amino acid sequences of plant invertases were obtained from the following sources, with their respective accession numbers enclosed in parentheses: *Vitis vinifera*, VvNI1, VvNI2 and VvNI3 (AM930846, GSVIVP00033188001, AM930850); *Beta vulgaris*, BvNI, BvVI, BvExINV1 and BvExINV2 (AJ422050, AJ425051, AJ422052, AJ422053); *Arabidopsis thaliana*, AthInvE, AthBFruc3 and AthBFruc4 (NM_122156, X11559, Y99111); ***Amaranthus hypochondriacus***, **AhN/AI1-6** (**JQ012920**, **JQ012922, isotig00661, isotig06148, isotig18198, isotig06149**; Délano-Frier et al. 2011, BMC Genomics 12: 363), **AhVI-1** (**JQ012921**), **AhCWI** (**JQ012923**); *Triticum aestivum*, TaAInv (AM295169); *Populus trichocarpa*, PtNI1 (Eugene 3.00190739); *Triticum monococcum*, TmVIN1 (AY575717); *Oryza sativa*, OsInv1 and OsInv2 (AP005738, AP004851); *Daucus carota*, DcS1 and DcInCW2 (X75352, M58362); *Vicia faba*, VfCWInv1, VfCWInv2 and VfVcInv (Z355162, Z35163, Z49831); *Chenopodium rubrum*, CrCIn1 and CrCIn3 (X81792, X81794); *Zea mays*, ZmInCW3, ZmInCW4 (AF043346, AF043347); *Hordeum vulgare*, HvCWInv1 (AJ534447); *Solanum tuberosum*, StInvCD111 and StInvCD141 (HQ197977, HQ110081) and *Lycopersicum esculentum*, SlWIV1 (AB004558).
