# Supplementary material for: Metabolic and enzymatic changes associated with carbon mobilization, utilization and replenishment triggered in grain amaranth (Amaranthus cruentus) in response to partial defoliation by mechanical injury or insect herbivory
Source: BMC Plant Biol. 2012 Sep 12;12:163. doi: 10.1186/1471-2229-12-163 (PMC3515461; doi:10.1186/1471-2229-12-163)
Supplement: Additional file 4 — Comparison of deduced amino acid sequences of plant ADP-glucose pyrophosphorylases (AGP). [file 1471-2229-12-163-S4.docx]

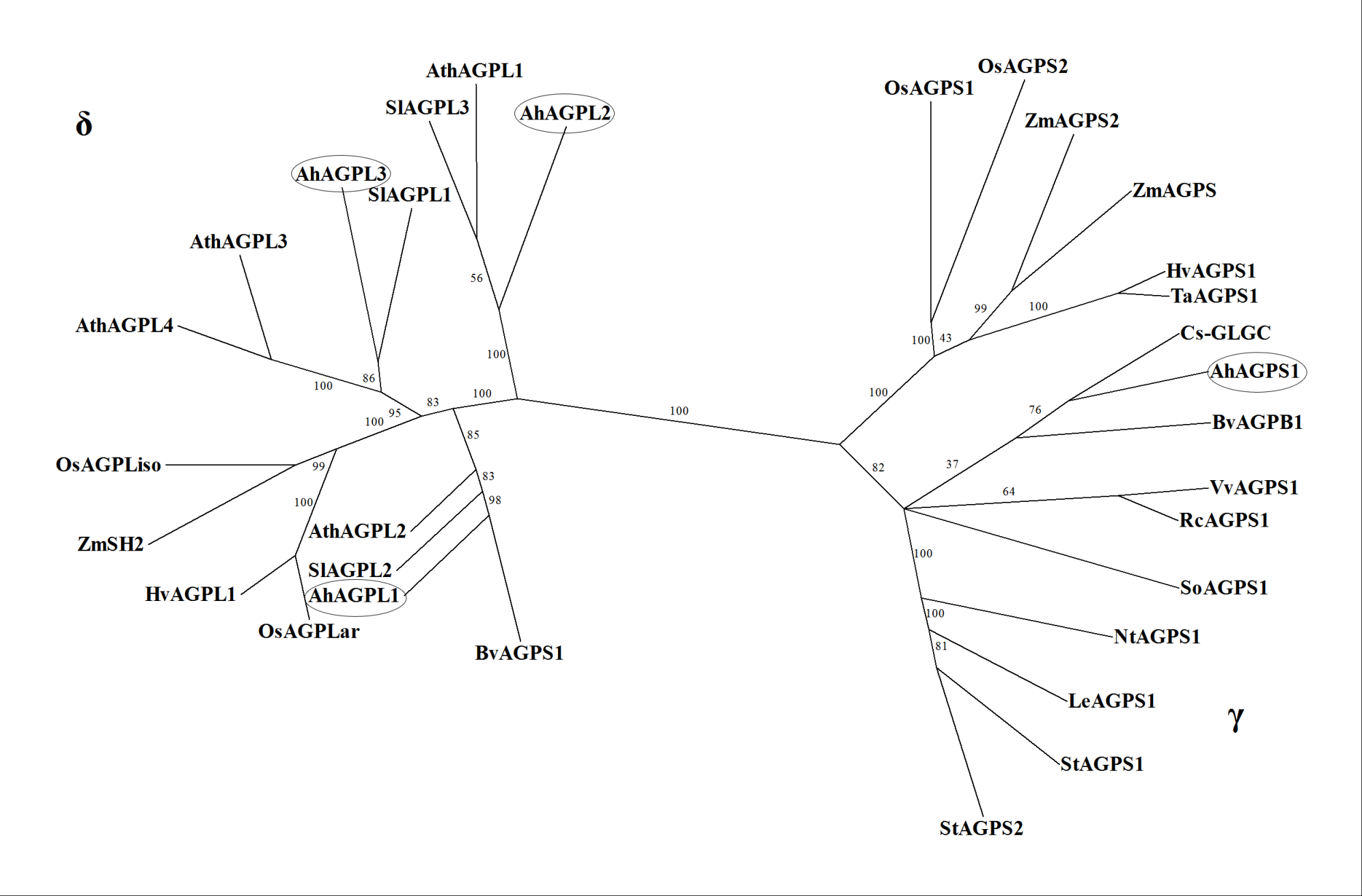


**Additional File 4.** Comparison of deduced amino acid sequences of plant ADP-glucose pyrophosphorylases (AGP). These are grouped into large (δ) and small (γ) subunits. Amaranth AGPs are encircled. The amino acid sequences were obtained from the following sources, with their respective accession numbers enclosed in parentheses: *Arabidopsis thaliana,* AthAGPL1, AthAGPL2, AthAGPL3 and AthAGPL4 (NM_121927, NM_102533, NM_120081, NM_127730); *Solanum lycopersicum,* SlAGPL1, SlAGPL2 and SlAGPL3 (U88089, U85496, U85497); *Lycopersicum esculentum*, LeAGPS1 (L41126.1); *Solanum tuberosum*, StAGPS1 and StAGPS2 (AY186620, X61186); ***Amaranthus hypochondriacus****,* AhAGPL-1, AhAGPL-2 and AhAGPL-3 (isotig09383, isotig14424, isotig19907; Délano-Frier et al. 2011, BMC Genomics 12: 363) and AhAGPS-1 (JQ034321); *Oryza sativa,* OsAGPLiso, OsAGPLar, OsAGPS1 and OsAGPS2 (GQ150819, GQ150833, NP_001061603, AK071826); *Zea mays,* ZmSH2, ZmAGPS and ZmAGPS2 (M81603.1, DQ118038, NP_001105038); *Hordeum vulgare*, HvAGPL1 and HvAGPS1 (FN179372, AAU06191); *Beta vulgaris*, BvAGPS1 and BvAGPB1 (x78900, X78899); *Triticum aestivum*, TaAGPS1 (FJ643609); *Citrus sinensis*, CsGLGC (DQ849076); *Vitis vinifera*, VvAGPS1 (XM_002263219); *Ricinus communis,* RcAGPS1 (XM_002524537); *Spinacia oleracea*, SoAGPS1 (X83500) and *Nicotiana tabacum*, NtAGPS1 (DQ399915).
