# Supplementary material for: Metabolic and enzymatic changes associated with carbon mobilization, utilization and replenishment triggered in grain amaranth (Amaranthus cruentus) in response to partial defoliation by mechanical injury or insect herbivory
Source: BMC Plant Biol. 2012 Sep 12;12:163. doi: 10.1186/1471-2229-12-163 (PMC3515461; doi:10.1186/1471-2229-12-163)
Supplement: Additional file 5 — Phylogenetic dendogram of known plant invertase inhibitors. (DOCX 77 kb) [file 1471-2229-12-163-S5.docx]

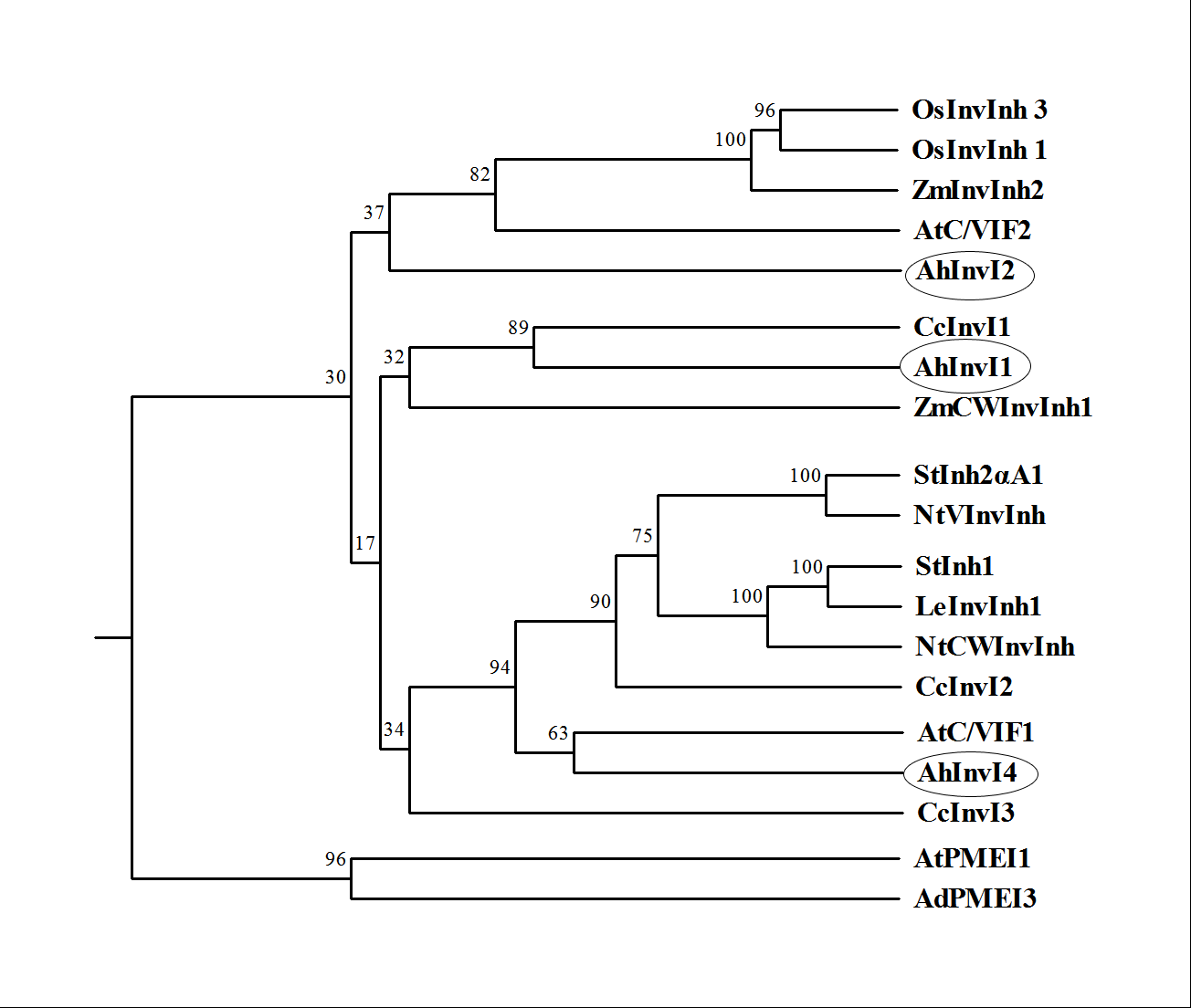


**Additional File 5.** Phylogenetic tree of reported plant invertase inhibitors. Amaranth invertase inhibitors are encircled. The deduced amino acid sequences were obtained from the following sources, with their respective accession numbers enclosed in parentheses: *Nicotiana tabacum*, NtCWInvInh and NtVInvInh (Y12805, CAA73334); *Solanum lycopersicum*, InvInh1 (NP_001234791); *Solanum tuberosum*, StInh1 and StInh2α-A1 (AY864819, FJ810205); *Coffea canephora*, CcInvI1, CcInvI2 and CcInvI3 (DQ834317, DQ834318, DQ834319); *Arabidopsis thaliana*, AtC/VIF1 and AtC/VIF2 (At1g47960, At5g64620); ***Amaranthus hypochondriacus*,** **AhInvI1, AhInvI12 and AhInvI14** (**isotig04350, isotig08065, isotig18063**; Délano-Frier et al. 2011, BMC Genomics 12: 363); *Zea mays*, ZmInvInh2 and ZmCWInvInh1 (AX21433 and Bate et al. 2004, Plant Physiol 134: 246-254); *Oryza sativa*, OsInvInh1 and OsInvInh3 (Rice Genome Annotation Project accession numbers AK288558 and AK070037); *Arabidopsis thaliana*, AtPMEI1 (At1g48020), and *Actinidia deliciosa* AdPMEI-3 (AB091090). This table expands information reported in Castrillón-Arbeláez and Délano-Frier 2011, Curr Enzyme Inhib 7: 169-177.
