# Supplementary material for: Metabolic and enzymatic changes associated with carbon mobilization, utilization and replenishment triggered in grain amaranth (Amaranthus cruentus) in response to partial defoliation by mechanical injury or insect herbivory
Source: BMC Plant Biol. 2012 Sep 12;12:163. doi: 10.1186/1471-2229-12-163 (PMC3515461; doi:10.1186/1471-2229-12-163)
Supplement: Additional file 6 — Structure of grain amaranth genes coding for a small subunit of ADP glucose pyrophosphorylase (AhAGPS-1) and a vacuolar invertase (AhVI-1). [file 1471-2229-12-163-S6.docx]

**(A)**

1000

**440**

**9**

**857**

**162**

**329**

**167**

**21**

PROMOTER

146

198

85

138

1015

600

I

II

III

IV

V

VI

VII

109

166

126

89

84

92

1048

372

PROMOTER

**225**

**297**

**270**

**180**

**104**

**112**

**99**

**168**

**196**

1551

I

II

III

IV

V

VI

VII

VIII

IX

**(B)**

Os -METRDDVADASALPYSYSPLPAGDAASADLAAA--R----RSRRRP--LCVALFLASAAVILAVAVLSGVRLAGR-PATTT--------

Sh -METRDTTAP---LPYSYTPLPAADAASAEVTGTGHRGGGGRSRRRS--LCAAALVLSAALLLAVAALAGVGVVD--PGVGT--------

Hv -MPTMDTTDR-----GSYAQLPDDAEAGS---AH---------RRRTGPLCAAILLTSAALLLAVAALAGVRVAGQLPVAGVIMSGQPTT

Ah MVESSDSILP-----YSYAPLAPTNNSNVEK----------QTRRRS--LKTGLLVFS-ALLISALIMNSVDFNTELNVTKNETKKLDDL

* ** * *** * * *

**(C)**

AhVI-1 WMNDPNGP WYHFFYQ FRDPTTAW WECVDFYP YASKTFYD KGWASIQA LGPFGL

AthBfruct4 WMNDPNGP WYHLFYQ FRDPTTAW WECVDFYP YASKSFYD KGWSSLQG LGPFGF

AthBfruct3 WMNDPNGP WYHFFYQ FRDPTTAW WECVDFYP YASKTFYD KGWSSVQG LGPFGF

BvVI WMNDPNGP WYHFFYQ FRDPTTAW WECVDFYP YASKTFYD KGWASLQG LGPFGL

VfVI WMNDPNGP WYHFFYQ FRDPTTAW WECVDFFP YASKTFYD KGWASVQS LGPFGL

OsInv2 WMNDPNGP WYHLFYQ FRDPTTAW WECVDFYP YASKTFYD KGWASVQS LGPFGI

DcS1 WMNDPNGP WYHLFYQ FRDPTTAW WECIDFYP YASKTFYD KGWASLQS LGPFGL

TmVIN1 WMNDPNGP WYHLFYQ FRDPTTAW WECIDFYP YASKTFYD KGWASLQS LGPFGL

**Additional File 6.** **(A)** Gene structure of vacuolar invertase (*AhVI-1*) and small subunit of ADP-glucose pyrophosphorylase (*AhAGPS-1*) (accession number: **JQ034321**) found in *Amaranthus hypochondriacus*. Exons are represented by solid black boxes and introns are represented by black lines, the lengths exons and introns are indicated in base pairs. **(B)** Amino-acid sequences corresponding to N-Terminal regions of vacuolar invertases from barley (Hv), rice (Os), sugarcane (Sh) and amaranth (Ah) with predicted motifs. Probable membrane-spanning domains are underlined. Possible YXXL motifs are shaded in gray and charged regions adjacent to membrane-spanning domains are boxed. Asterisks represent invariant amino-acids. **(C)** Alignment of well-conserved regions in known acid invertases. The gray shaded amino-acids show the four residues (Asp140, Asp265, Glu321 and Cys322) that conform the enzyme’s active site residues as proposed by Alberto et al. 2004, J Biol Chem 279: 18903-18910. The β-fructosidase motif (NDPD/NG) situated towards the N-terminus of the mature proteins is represented by dotted lines and the catalytic domain is represented by solid lines. The amino acid sequences were obtained from the following sources, with their respective accession numbers enclosed in parentheses: *Vitis vinifera*, VvNI1, VvNI2 and VvNI3 (AM930846, GSVIVP00033188001, AM930850); *Beta vulgaris*, BvVI (AJ425051); *Arabidopsis thaliana*, AthBFruc3 and AthBFruc4 (X11559, X9911); ***Amaranthus hypochondriacus***, **AhVI-1** (**JQ012921**); *Triticum monococcum*, TmVIN1, (AY575717); *Oryza sativa*, OsInv2 (AP004851); *Daucus carota*, DcS1 (X75352), and *Vicia faba*, VfVI (Z49831).
