# Supplementary material for: Metabolic and enzymatic changes associated with carbon mobilization, utilization and replenishment triggered in grain amaranth (Amaranthus cruentus) in response to partial defoliation by mechanical injury or insect herbivory
Source: BMC Plant Biol. 2012 Sep 12;12:163. doi: 10.1186/1471-2229-12-163 (PMC3515461; doi:10.1186/1471-2229-12-163)
Supplement: Additional file 7 — Regulatory elements identified in the promoter regions of the AhAGPS-1 and AhVI-1 genes. The 5’ regulatory region was analyzed using PLACE, PlantCARE and Genomatix Matinspector databases. [file 1471-2229-12-163-S7.docx]

**Additional File 7**. Regulatory elements identified in the promoter regions of the *AhAGPS-1* and *AhVI-1* genes. The 5’ regulatory regions were analyzed using PLACE, PlantCARE and Genomatix Matinspector databases.

| ***Cis*-acting elements Sequence** | | **Function** | | | **Gene** | |  |
| --- | --- | --- | --- | --- | --- | --- | --- |
| **A) Tissue-specific elements** | | | | | | |  |
| L1 box | TAAA | | L1 box, motif for L1 layer-specific expression. *Cis*-acting regulatory element involved in cotyledon development and epidermal cell differentiation. | AGPS, VI | | |  |
| AHBP | ATCA, ATTA, TAAT | | Arabidopsis homeobox protein. *Cis*-acting regulatory element involved in response to abscisic acid stimulus, maintenance of floral meristem identity and maintenance of inflorescence meristem identity. | AGPS, VI | | |  |
| MADS | AAAT, CCAT, TACC, TCCC, TTCC, CCAA, TGCT, ATAG,TACT, GAAA, GTAA, TACA, TATA, TCCT | | MADS box proteins. These factors regulate many developmental processes including flower, fruit and root development. | AGPS, VI | | |  |
| SWNS | TAAG, AAAG, CAAG | | Secondary wall NACS. *Cis*-acting regulatory element involved in anther dehiscence, lignin biosynthetic process and secondary cell wall biogenesis. | AGPS, VI | | |  |
| OPAQ | ATAT | | Opaque-2 like transcriptional activators. *Cis*-acting regulatory element involved in controlling the expression of distinct classes of endosperm genes. | AGPS, VI | | |  |
| LFYB | CCCA | | Plant specific floral meristem identity gene LEAFY (LFY). | AGPS, VI | | |  |
| SEF3-4 | TTTT, ACCC | | Soybean embryo factor 4. *Cis*-acting element involved in the regulation of seed proteins. | AGPS, VI | | |  |
| ASRC | TTGA | | AS1/AS2 repressor complex. *Cis*-acting regulatory element involved in leaf formation, DNA-dependent, negative regulation of transcription, petal development and proximal/distal pattern formation. | AGPS, VI | | |  |
| NCS1-2 | AAAT, TTCT, CTCG | | Nodulin consensus sequence 1 y 2. *Cis*-acting element involved in various functions of the nodule development and metabolism. | AGPS, VI | | |  |
| PALA | CCGT | | Conserved box A in *PAL* and *4CL* gene promoters. The elements appear to be necessary for elicitor- or light-mediated *PAL* gene activation. | AGPS, VI | | |  |
| STK motif | TAAA | | Storekeeper (STK). *Cis*-acting regulatory element important for tuber-specific and sucrose-inducible gene expression. | AGPS | | |  |
| CAR motif | AAGA, AACA | | CA-rich motif. *Cis*-acting element involved in the regulation of seed proteins. | AGPS | | |  |
| **B) Light-responsive elements** | | | | | |  | |
| CCAF | AATC, AATA | [Circadian control factors. *Cis*-acting element involved in long-day photoperiodism and flowering](http://www.genomatix.de/cgi-bin/matbase/matbase.pl?s=d7f0d10537be7aa197b7b0792eb5fa35;NAME=GOID_d5ac698bef0ffd5fc8215166bee3c6b5edb2e85b6dd4e9ca;ML=84). | | AGPS, VI | | |  |
| I box | GATA | Light box Element. *Cis*-acting regulatory element conserved in sequence upstream of light-regulated genes. | | AGPS, VI | | |  |
| LRE motif  GAP box | ATCT | Light responsive element motif, not modulated by different light qualities.  Light box element. | | AGPS, VI  AGPS, VI | | |  |
| **C) Component-specific elements** | | | | | | |  |
| SUC box | AAAT, ATAT | *Cis*-acting regulatory element required for sugar responsive gene expression. | | AGPS, VI | | |  |
| PSRE | GAAA | Pollen-specific regulatory elements. | | AGPS | | |  |
| LEG box | GCAT, CATG | Positive regulator of germination. | | AGPS | | |  |
| ROOT | CAAG | Root hair-specific *cis*-elements in angiosperms. | | AGPS | | |  |
| GAGA | AGAG | *Cis*-acting regulatory element involved in the development of ovules. | | AGPS | | |  |
| TERE | AAAG | Tracheary-element (TE)-regulating *cis*-elements, conferring TE-specific expression. | | VI | | |  |
| PSPE | GAAG | Protein secretory pathway element. | | VI | | |  |
| PREM | CGAC; CGAT | Motifs of plastid response elements. | | VI | | |  |
| **D) Binding-site-specific elements** | | | | | | |  |
| DOFF | AAAG | DNA binding with one finger (DOF) factor. *Cis-*acting regulatory element involved in light-regulated gene expression and of the phloem-specific Dof zinc finger transcription factor, a negative regulator in both the phytochrome A (phyA) and phytochrome B (phyB) signaling pathways. | | AGPS, VI | | |  |
| CAAT | CCAA | CCAAT binding factors. Transcriptional regulator. | | AGPS, VI | | |  |
| HMGF | TATT | High mobility group factors. Elements in *cis* that recognize HMG proteins. These proteins can enhance the structural flexibility of DNA, facilitating the assembly of nucleoprotein structures that control various DNA-dependent processes, such as transcription and recombination. | | AGPS, VI | | |  |
| GT box | ATGG, TTAA, GTAA, GTTA | *Cis*-acting element involved in the transcriptional repression of ribosomal protein cS1 in non-photosynthetic tissues, such as roots. | | AGPS, VI | | |  |
| **E) Regulation-specific elements** | | | | | | |  |
| TEFB | ACGG | *Cis*-acting regulatory element involved in the transcriptional activation of plant genes that are overexpressed in cycling cells. | | VI | | |  |
| HOCT | ATCC | Octamer motif found in plant histone H3 and H4 genes. Elements conferring S phase-specific transcriptional activation. | | VI | | |  |
| E2FF | TTCC | E2F-homolog cell cycle regulators. E2F-like sites in the tobacco PCNA promoter function as positive *cis*-elements responsible for the expression in actively dividing cells. | | AGPS | | |  |
| CDC5 | AGCG | *Cis*-acting regulatory element may function in cell cycle regulation. | | AGPS | | |  |
| MSAE | AACG | M-phase-specific activator elements. | | AGPS | | |  |
| TATC-box | TATCCCA | *Cis-*acting element involved in gibberellin-responsiveness. | | AGPS | | |  |
| GARE | AAACAGA | Gibberellin-responsive element. | | AGPS | | |  |
| **F) Stress-specific elements** | | | | | | |  |
| MYB  (MYBS, MYBL, MIIG) | ATCC, CTGT, GTTA, ATAT, TAGT, AAAT, CGGT, AGTT | *Cis*-acting regulatory element involved in anthocyanin and flavonoid production, and in trichome differentiation. These elements also regulate genes that are responsive to water stress, development and seed germination. | | AGPS, VI | | |  |
| OCSE | GACG | Enhancer element first identified in the promoter of the octopine synthase gene (OCS) of the *Agrobacterium tumefaciens* T-DNA. *Cis*-acting regulatory element involved in defense response to bacteria. | | AGPS, VI | | |  |
| RAV5 | AACA | *Cis*-acting regulatory element involved in defense and senescence of plants. | | AGPS, VI | | |  |
| ERSE | CACA | ER stress-response elements. Element involved in the correct assembly and folding of proteins synthesized in the endoplasmic reticulum. In plants, the importance of the ER stress response has been implicated in the development and function of pollen and tapetal cells, as well as plant-specifics cellular process, such as pathogen response and seed development. | | AGPS, VI | | |  |
| W box | TTGA | *Cis*-acting regulatory element involved in direct fungal elicitor stimulated transcription of defense genes and activation of genes involved in response to wounding. | | AGPS, VI | | |  |
| HSE | AGAA, ATTC, CTTC | Heat shock factors. | | AGPS, VI | | |  |
| ARE | TGGTTT | Anaerobic Response Element. *Cis*-acting regulatory element essential for induction under anaerobic conditions. | | AGPS, VI | | |  |
| TC-rich repeats | ATTTTCTCCA | *Cis*-acting element involved in defense and stress responsiveness. | | AGPS, VI | | |  |
| TGACG-motif | TGACG | *Cis-*acting regulatory element involved in methyl jasmonate (MeJA)-responsiveness. | | AGPS | | |  |
| WNAC | AAACG | Wheat NAC-domain DNA binding factor. *Cis*-acting regulatory element involved in embryonic, floral and vegetative development, lateral root formation and auxin signaling. This element has also been found in genes related to defense. | | AGPS | | |  |
| G box | ACGT | *Cis*-acting regulatory element involved in defense response, incompatible interaction, photomorphogenesis, response to blue light, response to xenobiotic stimuli, in the salicylic acid mediated signaling pathway and in systemic acquired resistance. | | AGPS | | |  |
| FORC | GGGC | Fungal and oomycete pathogen response cluster - promoter motif. *Cis*-acting regulatory element that integrates stimuli related to defense- and light-signaling. | | AGPS | | |  |
| GARP | AGAT | Myb-related DNA binding proteins (Golden2, ARR, Psr). *Cis*-acting regulatory element involved in cytokinin mediated signaling pathway, primary root development, regulation of anthocyanin and chlorophyll biosynthetic processes, regulation of circadian rhythm, responses to cytokinin stimulus and stress, and shoot development. | | AGPS | | |  |
| VRES | GCTG | [VIP1 responsive elements. *Cis*-acting regulatory element involved in regulation of response to stress](http://www.genomatix.de/cgi-bin/matbase/matbase.pl?s=2e6cffd5dde20bd44ea7cf108d219ae0;NAME=GOID_7deb90f0adfcc85bb011e18022bc99de8c4575e7c2a24b36;ML=84). | | AGPS | | |  |
| GCCF | CGCC | GCC box family. *Cis*-acting regulatory element involved in ethylene mediated signaling pathway, JA mediated signaling pathway, and in DNA-dependent positive regulation of transcription. | | AGPS | | |  |
| AGP1 | GATC | AG-motif binding protein 1. Element sufficient to confer responsiveness to wounding and elicitor treatment. | | VI | | |  |
| ERE | TGGA | Ethylene responsive element. *Cis*-acting regulatory element involved in defense response to bacteria, ethylene and sugar mediated signaling pathways. | | VI | | |  |
| SLIM | CGTT | Sulfur limitation, elements found in genes inducible during sulfur deprivation. | | VI | | |  |
| DREB | ACCG | Dehydration responsive element binding factors. *Cis*-acting regulatory element involved in responses to cold- and salt-stress, and to water deprivation. | | VI | | |  |
| JERE | AGACCGCC | JA- and elicitor-responsive element. | | VI | | |  |
| ELI-box3 | AAACCAATT | Elicitor-responsive element. | | VI | | |  |
