# Supplementary material for: Metabolic and enzymatic changes associated with carbon mobilization, utilization and replenishment triggered in grain amaranth (Amaranthus cruentus) in response to partial defoliation by mechanical injury or insect herbivory
Source: BMC Plant Biol. 2012 Sep 12;12:163. doi: 10.1186/1471-2229-12-163 (PMC3515461; doi:10.1186/1471-2229-12-163)
Supplement: Additional file 8 — Comparison of the 5’ regulatory regions identified in the ADP-glucose pyrophosphorylase and vacuolar invertase genes of Solanum tuberosum, S. lycopersicum, Beta vulgaris and Amaranthus hypochondriacus. [file 1471-2229-12-163-S8.docx]

**Additional File 8.** Comparison of the 5’ regulatory regions identified in the ADP-glucose pyrophosphorylase and vacuolar invertase genes of *Solanum tuberosum* (GenBank, L36648), *S. lycopersicum* (SolGenomics, Solyc08g079080), *Beta vulgaris* (AJ277455) and *Amaranthus hypochondriacus*. The items displayed are unique to each species’ promoter and are involved in development and in different stress responses (items in bold). (*) The sequence reported for *S. tuberosum* is 712 bp shorter than that of amaranth. Most probably, these elements will be detected once the promoter region of this gene is completed.

| **Gene** | **Plant** | **Cis-acting elements** |
| --- | --- | --- |
| *AGPS* | *Solanum tuberosum* | TDFF, AREF, EPFF, TEFB, URNA, BRRE, SBPD, IDDF, NACF |
|  | *Amaranthus hypochondriacus* | GCCF, PALA, MIIG, E2FF, ASRC, RAV5*, EREF*, CAAT, ERSE, MYBS, ROOT, VRES*, WBXF, WNAC, SEF4, FORC^A^*, GAGA, CDC5, LEGB, NCS2, CNAC, LFYB, GARP. |
| *InvVI-1* | *Beta vulgaris* | ABRE, IDDF, NCS1, WNAC, LEGB, AREF, GAGA, STKM, GBOX, PNRE, CGCG, PSRE |
|  | *Amaranthus hypochondriacus* | ERSE, PSPE, AGP1, HOCT, SEF3, CARM, PREM, SLIM, RAV5, MYCL, JERE, TC-rich repeats. |
|  | *Solanum lycopersicum* | ABRE, MSAE, NCAC, HEAT, TCPF, PSRE, DPBF, TELO, ROOT, E2FF, NCS1, LEGB, WNAC, GARP, GBOX |
