# Supplementary material for: Metabolic and enzymatic changes associated with carbon mobilization, utilization and replenishment triggered in grain amaranth (Amaranthus cruentus) in response to partial defoliation by mechanical injury or insect herbivory
Source: BMC Plant Biol. 2012 Sep 12;12:163. doi: 10.1186/1471-2229-12-163 (PMC3515461; doi:10.1186/1471-2229-12-163)
Supplement: Additional file 9 — Results of an atypical experiment showing a long-term accumulation of starch in stems and roots of MD plants. [file 1471-2229-12-163-S9.docx]

**A**

**B**

**C**

**D**


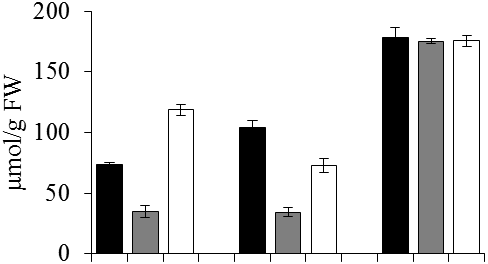


1 5 30 dppd

**

***

***

*

C

HD

MD


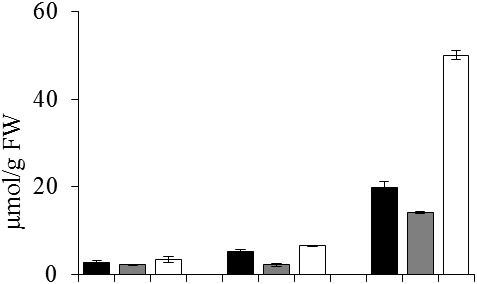


1 5 30 dppd

**

***

*

1 5 30 dppd


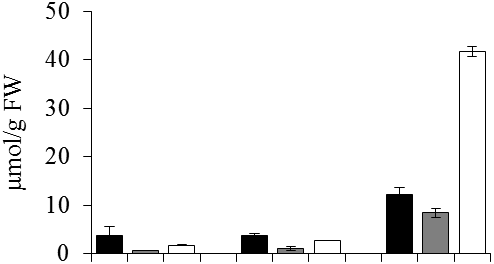


**

***

30 dppd


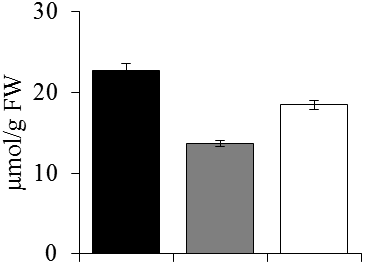


**

*

**Additional File 9.** Atypical long-term starch accumulation in roots and stems of defoliated grain amaranth plants. Starch levels were measured at different days post partial defoliation (dppd) in (**A**) source leaves^1^, (**B**) stems, (**C**) roots and (**D**) panicles of intact control and defoliated *Amaranthus cruentus* plants. Defoliation was produced either by insect herbivory (**HD**) or mechanical damage (**MD**). Data represent means ± standard error of three replicates of a specific experiment. Asterisks indicate significant difference from controls at *P < 0.05; **P < 0.01; ***P < 0.001. ^1^In defoliated plants, all three source leaves sampled were damaged.
