# Supplementary material for: Metabolic and enzymatic changes associated with carbon mobilization, utilization and replenishment triggered in grain amaranth (Amaranthus cruentus) in response to partial defoliation by mechanical injury or insect herbivory
Source: BMC Plant Biol. 2012 Sep 12;12:163. doi: 10.1186/1471-2229-12-163 (PMC3515461; doi:10.1186/1471-2229-12-163)
Supplement: Additional file 10 — Fatty acid composition in roots of control and defoliated grain amaranth plants. [file 1471-2229-12-163-S10.docx]

**Additional File 10.** Fatty acid content in roots of control and defoliated grain amaranth plants. Individual fatty acids were measured at different days post partial defoliation (dppd) in roots of intact control (**C**) and defoliated *Amaranthus cruentus* plants. Defoliation was produced either by insect herbivory (**HD**) or mechanical damage (**MD**). Data represent means ± standard error of three replicates of a representative experiment that was replicated twice.

|  | **dppd** |  | **C16: 0^1^** | **C18: 0** | **C18: 1** | **C18: 2** | **C18: 3** |
| --- | --- | --- | --- | --- | --- | --- | --- |
|  | 1 | C | 4.28 + 1.102 | 0.30 + 0.045 | 1.92 + 0.617 | 7.49 + 1.478 | 0.82 + 0.330 |
|  |  | HD | 3.72 + 0.679 | 0.25 + 0.021 | 2.19 + 0.419 | 6.07 + 0.617 | 0.56 + 0.191 |
|  |  | MD | 3.34 + 0.638 | 0.67 + 0.507 | 1.92 + 0.391 | 6.45 + 0.907 | 0.60 + 0.228 |
|  | 5 | C | 1.77 + 0.075 | 0.16 + 0.004 | 0.74 + 0.027 | 3.21 + 0.239 | 0.31 + 0.057 |
|  |  | HD | 3.13 + 0.088^***^ | 0.28 + 0.043^*^ | 1.94 + 0.099^***^ | 5.72 + 0.451^**^ | 0.54 + 0.084 |
|  |  | MD | 2.95 + 0.133^**^ | 0.22 + 0.022^*^ | 1.60 + 0.120^**^ | 5.47 + 0.288^**^ | 0.48 + 0.079 |
|  | 30 | C | 2.36 + 0.791 | 0.13 + 0.087 | 1.33 + 0.621 | 3.43 + 0.166 | 0.29 + 0.074 |
|  |  | HD | 1.50 + 0.131 | 0.17 + 0.065 | 0.96 + 0.039 | 2.48 + 0.244 | 0.15 + 0.062 |
|  |  | MD | 1.45 + 0.070 | 0.09 + 0.005 | 0.89 + 0.013 | 2.78 + 0.270 | 0.21 + 0.080 |

^1^In µg/mg

Asterisks indicate significant difference from controls at *P < 0.05; **P < 0.01; ***P < 0.001.
