# Supplementary material for: Metabolic and enzymatic changes associated with carbon mobilization, utilization and replenishment triggered in grain amaranth (Amaranthus cruentus) in response to partial defoliation by mechanical injury or insect herbivory
Source: BMC Plant Biol. 2012 Sep 12;12:163. doi: 10.1186/1471-2229-12-163 (PMC3515461; doi:10.1186/1471-2229-12-163)
Supplement: Additional file 11 — Primers used for gene expression analysis by qRT PCR. [file 1471-2229-12-163-S11.docx]

**Additional File 11**. Primers used for gene expression analysis by qRT PCR.

| **Gene** |  | **Sequence** |
| --- | --- | --- |
| *AhAGPS-1^1^* | Forward | CGGTGAAGGCTGTGTGATAAAGAACTGC |
|  | Reverse | ATGAGTGTTCTTCCCGATTCCAATAGGT |
| *AhAGPL-1^2^* | Forward | AAGCCAGTCATATTTGCAGAGTTT |
|  | Reverse | ATTGTCGAGTCCATCAGTTAATAA |
| *AhAGPL-2^3^* | Forward | AGAAGCTTCCATGAGATACAATAGA |
|  | Reverse | ATGATTATTGGGAAGATATTGGAACTA |
| Granule-Bound Starch Synthase (*AhGBSS*) | Forward | ACCTCAAGTCCATGTATCAATCTAA |
|  | Reverse | CCAGTTGATTTTCCTTCCTTTAA |
| Soluble starch synthase class III (*AhSS-III*) | Forward | GAGTATATGGAGCTTTATCACGC |
|  | Reverse | CCCACATTTACTCTACATTTTCC |
| Soluble starch synthase class IV (*AhSS-IV*) | Forward | AACTCATCATATTAAGGGGCTCT |
|  | Reverse | CAAAATTGGCAACATCACTTACTA |
| β-amylase 1 (*AhBMY-1*) | Forward | GAGCTTACAAGTCACAATACCAC |
|  | Reverse | GCAAGCTCAAAAGGTTTATG |
| Vacuolar invertase (*AhVI-1*) | Forward | CAGGATCGGTTGTGCACTTGGATGTCA |
|  | Reverse | ATCATCAGCAAGAACCAGAAGACCGA |
| Neutral/alkaline invertase-1 (*AhN/AI-1*) | Forward | ATATTGCTTCTTCATTGATGTTTG |
|  | Reverse | TAATTTTATCGAACAATGTCTTTTGAA |
| Invertase Inhibitor-1 (*AhInvI-1*) | Forward | CGATATAAGCGTAAAACTGGAAGAGA |
|  | Reverse | CTTCTCCTTAAACCCTGCTTCACATACAT |
| Invertase Inhibitor-2 (*AhInvI-2*) | Forward | TGTGGAGCTGTCGATGTGGAACTTC |
|  | Reverse | AAATCCGTCTTCTGCATCCTAATTCCG |
| Invertase Inhibitor-4 (*AhInvI-4*) | Forward | CACTCTCGTACGACCACCACCG |
|  | Reverse | GGTGGACGTGGTTAAAACAGGGTATTC |
| Sucrose synthase-1 (*AhSuSy-1*) | Forward | CCGTCGTGAGGCTCGTCGTTACC |
|  | Reverse | CATCGTACAATCAGTGCACATTC |
| Sucrose synthase-2 (*AhSuSy-2*) | Forward | GAAATCCGTACCTCTGGCATC |
|  | Reverse | CACTATACAAAACAAGCAGGAAAATA |
| Vacuolar processing enzyme gamma (*Ah*γ*VPE*) | Forward | AATGGTGAAGACGTTTATAATGGAGTT |
|  | Reverse | GGAAGTGGAAAAGTCGTCGATAG |
| *AhSnRK-1^4^* | Forward | TCTCGCAAAACATCTGTCATAG |
|  | Reverse | CTGGAAGTTATGCAAAAGTGGTT |
| Sucrose phosphate synthase (*AhSPS*) | Forward | AACAATGCATCAAAACAAAGATAT |
|  | Reverse | ATGATACGCGATAAGACACTG |
| Glucose-6P/ phosphate transporter (*AhGPT*) | Forward | ATCGAAAGGTCTTGTTTACATCA |
|  | Reverse | ATTTCCATTTCCACAAGATTTTAT |
| Sucrose transporter  (*AhSUT-1*) | Forward | CTCGACGTAGCCAACAACACTCTTC |
|  | Reverse | GTGAATGGGAATATTTTGTAGAGACGG |
| *AhPPT^5^* | Forward | GACAAACACTAACCAAAACTAAGCGGC |
|  | Reverse | ACTGTGTGAAGAGGGTGGTTGTC |
| Kunitz trypsin inhibitor (*AhKTI*) | Forward | TTACCACAGCCATCCTTCTCAT |
|  | Reverse | GAGGGTCTTGTTGTTGGAGTTAC |
| Lypoxygenase 2 (*AhLOX2*) | Forward | ATCGGTGCGTTGTTTTCCT |
|  | Reverse | CACTGTCTGTTGATGCTATTGC |
| Senescence-associated gene (*AhSAG*) | Forward | TGGGTTTTCTTCTCCTCTCACTATAA |
|  | Reverse | CGATGATCAAGATATTTACGTAATTTG |
| Actin (*ACT)* | Forward | CGTGACCTGACTGATTACCTTA |
|  | Reverse | GCTCGTAGTTCTTCTCAATGGC |
| β-Tubulin *(TUB)* | Forward | TCTCAGCAGTATGTCTCCCTCA |
|  | Reverse | TCTACTTCTTTGGTGCTCATCTT |
| Elongation factor 1α (*EF1α*) | Forward | GCCAAATATCTAAGAAACAAATGC |
|  | Reverse | TAGCACAACCACATGATATTTCTT |

^1^*AhAGPS-1*: Plastidial ADP-glucose pyrophosphorylase, small sub-unit 1. ^2^*AhAGPL-1*: ADP-glucose pyrophosphorylase large sub-unit 1. ^3^*AhAGPL-2*: Plastidial ADP-glucose pyrophosphorylase large sub-unit 2. ^4^Sucrose-non-fermentation1 (Snf1)-related protein kinase 1. ^5^*AhPPT*: Phosphoenolpyruvate/phosphate transporter.
