# Supplementary material for: Metabolic and enzymatic changes associated with carbon mobilization, utilization and replenishment triggered in grain amaranth (Amaranthus cruentus) in response to partial defoliation by mechanical injury or insect herbivory
Source: BMC Plant Biol. 2012 Sep 12;12:163. doi: 10.1186/1471-2229-12-163 (PMC3515461; doi:10.1186/1471-2229-12-163)
Supplement: Additional file 12 — Primers used to amplify the 5 'and 3' cDNA ends (RACE) and the genomic sequences, including part of the promoter regions, of the AhVI-1 and AhAGPS-1 genes. [file 1471-2229-12-163-S12.docx]

**Additional File 12.** Primers used to amplify the 5 'and 3' cDNA ends (RACE) and to obtain the genomic sequences, including part of the promoter regions, of the *AhVI-1* and *AhAGPS-1* genes.

|  | **Gene** | **Sequence** | |
| --- | --- | --- | --- |
| RACE | *AhVI-1* | | Race 5’ CGCCGTTGAAGTCGTACCATTGG  Race 3’ AAAGCGGGTATGGATGATGATAGGAATGA |
|  | *AhCWI* | | Race 5’ CCTGTGAAAAGAATTGTGGGTTTGGATCC |
|  | *AhAGPS-1* | | Race 5’ ATGAGTGTTCTTCCCGATTCCAATAGGT  Race 3’ GACCGTTCATCTCCAATCTATACAC |
|  | *AhN/AI-1* | | Race 5’ GGCATCAGTTGAATATTCCTCCGTTTTG  Race 3’ GAGTCAGCCATTGGTCGCGTG |
|  | *AhN/AI-2* | | Race 5’ AAAGCAGTTGATATGGCAGAGAAG  Race 3’ GGTCAGGGCACAAACACATATCTC |
|  | *AhSuSy-1* | | Race 5’ TCACCCCAGCCTCTTTCCAAACC  Race 3’ GCCGTTTACTGCCTCTTTCCCTCG |
|  | *AhSuSy-2* | | Race 5’ CACTATACAAAACAAGCAGGAAAATA  Race 3’ CCTGGCAAAAGTATTCCGAAAG |
| GENOME WALKER | *AhVI-1* | | G.W. 5’  TTTGGACTTGGACTGATTCATTGGTTG,  CGCCGTTGAAGTCGTACCATTGG, CTCCCCAAAATTAATTTCACAAAACAAA, GCAACTTACCTTTTACCAAACTACAATCTC,  TTCTCCGACACACCATTAGGCTTACCAC, AACCTTAAACAACAACTCATTTGATCC, ATGAAAGAAAAGTGAAGGGAAAATGGCAA, ACAGAGGAAGGAAGAAAAACATTGGTC  G.W.3’  GGATCAAATGAGTTGTTGTTTAAGGTT, TGGTAAGCCTAATGGTGTGTCGGAGAA, GCAGGATCGGTTGTGCACTTGTATGTCA, ATGGAATTTGATCGGGTTTGTGTGCAGT, TCAATCATAGAAGCGTTTGCACAAGG, CCAACCAAGGCGATATACAGAGATGC,  TTCTTCATGCTGTGCCTGGTACTGGTAT, AAAGCGGGTATGGATGATGATAGGAATGA |
|  | *AhAGPS-1* | | G.W. 5’  TTTGGACTTGGACTGATTCATTGGTTG,  CGCCGTTGAAGTCGTACCATTGG,  TGGAAATGCGCAATATGAACCGT,  TGAATAGTGCACGGTGGCCAAATGT, CGGATAGAGAAGAAGAGGAGAAAGCGA, ATGAGTGTTCTTCCCGATTCCAATAGGT, CATCGGTTTCTCTCGCGGCTTCTTGTAC, TACCAATATCTTCCCAATAACCATCATAC, ACACTCTGACATCACAAACACACATCGT, CCCTCGTTCTTGTAGCCTCCCATGTTG  G.W. 3’:  TTCTTCATGCTGTGCCTGGTACTGGTAT, AAAGCGGGTATGGATGATGATAGGAATGA, TCGCTTTCTCCTCTTCTTCTCTATCCG, GAATGCTCGTATTGGGGATGATGTCA, ATAATGTACAAGAAGCCGCGAGAGAAACC, AAGGAGAACAACTGAAAGCGATGA, |
